# Biosynthesis of oxygen and nitrogen-containing heterocycles in polyketides

**DOI:** 10.3762/bjoc.12.148

**Published:** 2016-07-20

**Authors:** Franziska Hemmerling, Frank Hahn

**Affiliations:** 1 Institut für Organische Chemie and Zentrum für Biomolekulare Wirkstoffe, Gottfried Wilhelm Leibniz Universität Hannover, Schneiderberg 38, 30167 Hannover, Germany; 2 Fakultät für Biologie, Chemie und Geowissenschaften, Universität Bayreuth, Universitätsstraße 30, 95440 Bayreuth, Germany

**Keywords:** biosynthesis, chemoenzymatic synthesis, enzymology, heterocycles, polyketides

## Abstract

This review highlights the biosynthesis of heterocycles in polyketide natural products with a focus on oxygen and nitrogen-containing heterocycles with ring sizes between 3 and 6 atoms. Heterocycles are abundant structural elements of natural products from all classes and they often contribute significantly to their biological activity. Progress in recent years has led to a much better understanding of their biosynthesis. In this context, plenty of novel enzymology has been discovered, suggesting that these pathways are an attractive target for future studies.

## Introduction

### Heterocycles

Heterocycles are important structural elements, which are present in natural products from all classes and also in many biologically active synthetic compounds. They often contribute significantly to their structural and physical properties as well as to their biological activity [1–3]. Heterocycles can for example be involved in cation complexation as known for ionophoric polyethers or introduce conformational rigidity into a molecule, which is crucial for target binding [4].

Oxygen heterocycles are mainly found in carbohydrates, polyketides, peptides and terpenoids. Nitrogen heterocycles are part of peptides and alkaloids. Both can of course also occur in the respective hybrid natural products. Sulphur-containing heterocycles are present in few polyketides and more widespread in peptidic natural products of both, non-ribosomal and post-ribosomal modified origin [5].

The biosynthetic mechanisms for heterocycle formation are numerous, and range from simple addition or condensation reactions to oxidative ring closures. The large number of mechanistically different cyclisation modes triggers the interest on the responsible enzymes. Due to the relevance of heterocycles, understanding the enzymology of heterocycle formation is also an important milestone on the way to using the enzymes as chemoenzymatic tools in natural product synthesis and medicinal chemistry [6–7].

### Polyketides

Polyketide natural products are biosynthesised by polyketide synthases (PKSs) of the types I–III. Type I PKS are multimodular megaenzyme complexes that produce linear, reduced polyketides in an assembly line process that uses acyl carrier proteins (ACP), ketosynthase (KS) and acyl transferase (AT) domains as well as ketoreductase (KR), dehydratase (DH), enoyl reductase (ER) and thioesterase (TE) domains [6,8]. The PKS intermediates remain tethered to the megaenzyme via a thioester linkage during the whole process.

**Scheme 1:**
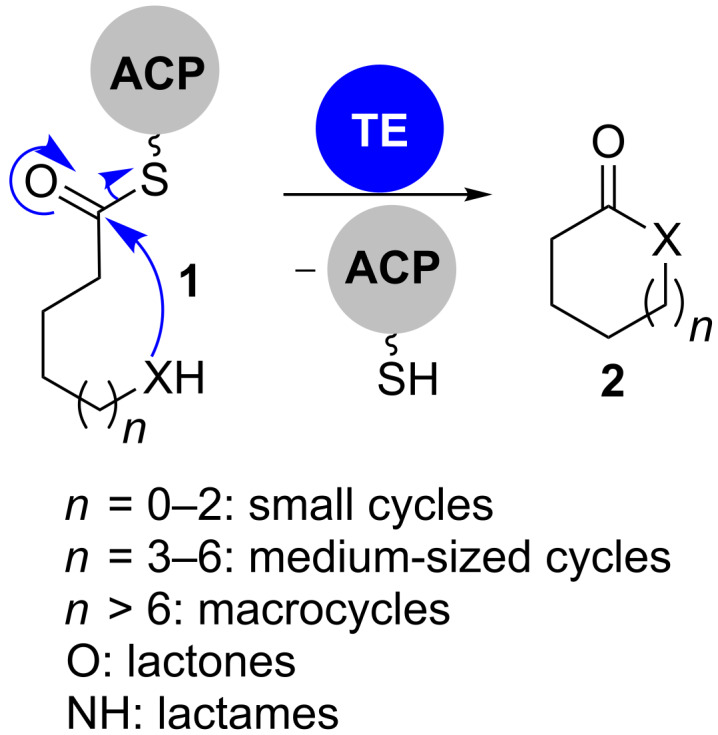
Schematic description of the cyclisation reaction catalysed by TE domains. In most cases, the nucleophile “X” represents oxygen or nitrogen leading to lactones or lactams, respectively.

Among these domains, only TE domains participate in cyclisation reactions as part of their standard catalytic repertoire (Scheme 1). They transacylate the thioester of a PKS-bound polyketide onto a nucleophile. If the nucleophile is water, this leads to carboxylic acids. The reactions of backbone hydroxy groups or amines consequently give lactones and lactams. TE domains mostly form macrocycles or more rarely medium-sized and small cycles with defined size.

Type II and type III PKS are mono-modular and form aromatic structures. Their gene clusters can contain additional cyclase/aromatase domains and a chain-length factor that together force particular folding patterns of a polyketone precursor and thus particular ring systems [9–11].

Most heterocycles in polyketides are formed by specialised PKS domains and tailoring enzymes. These can be active during assembly of the nascent PKS precursor (as for example in the case of pyran/furan formation via oxa-Michael addition, see chapters 1.1.1 and 1.2.1), during the cleavage of the fully elongated precursor from the PKS (as for example for tetronates, tetramates and pyridinones, see chapters 1.7.1, 2.2.1 and 2.1.3) or during post-PKS tailoring (as for example during oxidative cyclisation in aureothin biosynthesis, see chapter 1.2.2).

This review intends to give an overview on the mechanisms involved in heterocycle formation during polyketide biosynthesis. A focus will be placed on oxygen and nitrogen-containing heterocycles due to their abundance and relevance.

Although the genuine polyketide biosynthesis machinery does not harbour enzymatic units that introduce nitrogen, we expanded the scope of this article to those products of polyketide synthase–non ribosomal peptide synthetase (NRPS) hybrid systems in which the polyketide portion strongly dominates the overall structure and in which the amino acid nitrogens are incorporated into the respective heterocycles.

We will not cover medium-sized and macrocyclic lactones and lactams, but concentrate on small heterocycles with ring sizes between 3 and 6 atoms (for a review about macrolactones see reference [12]).

## Review

### 1 Oxygen-containing heterocycles

**Scheme 2:**
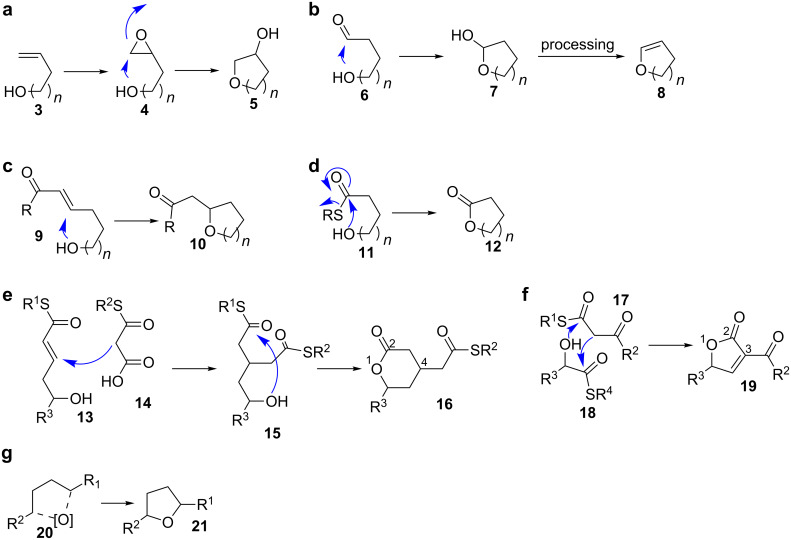
Mechanisms for the formation of oxygen heterocycles. The degree of substitution can differ from that shown in the scheme. In b, other modes of processing are possible in the second step. Partially redrawn from [13].

Oxygen-containing heterocycles are biosynthesised in seven principal ways (Scheme 2). Those comprise nucleophilic addition of a hydroxy group to electrophiles like epoxides **4**, carbonyl groups **6** or Michael acceptors **9**, potentially followed by further processing (a–c in Scheme 2).

Lactones **12** are formed by transacylation of a thioester to a hydroxy group (d in Scheme 2). A Michael addition–lactonisation cascade leads to pyranones with a substituent in the 4-position **16** (e in Scheme 2). 3-Acylfuran-2-ones (**19**, 3-acyltetronates) are formed by acylation–Dieckmann condensation between 2-hydroxythioesters **18** and β-ketothioesters **17** (f in Scheme 2). The oxidative cyclisation after C–H activation of alkyl carbons is known for the formation of furan rings **21** (g in Scheme 2).

#### 1.1 Pyrans

**Scheme 3:**
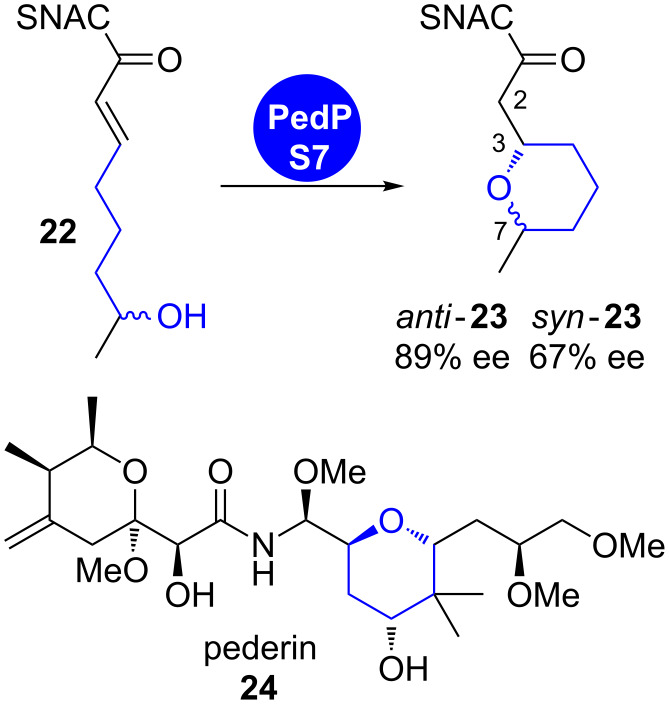
Pyran-ring formation in pederin (**24**) biosynthesis. Incubation of recombinant PedPS7 with substrate surrogate **22** gave conversion into cyclic stereoisomers *anti*-**23** and *syn*-**23** [14].

**Scheme 4:**
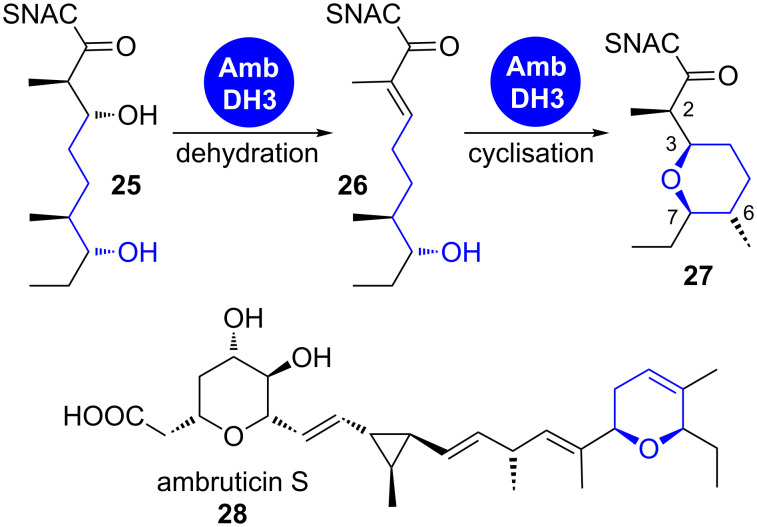
The domain AmbDH3 from ambruticin biosynthesis catalyses the dehydration of **25** and subsequent cyclisation to tetrahydropyran **27** with high stereoselectivity [15].

**1.1.1 oxa-Michael addition:** The oxa-Michael addition on an α,β-unsaturated thioester intermediate leads to oxygen heterocycles along with the formation of up to two new stereocentres. Its appearance in several polyketide biosynthetic pathways was proposed for a decade based on gene cluster analysis. An in vitro characterisation of responsible catalytic units has however only recently been achieved. Two pyran-forming cyclase domains were characterised in the pederin (**24**) and the ambruticin (**28**) biosynthetic pathways (Scheme 3 and Scheme 4) [14–15].

PedPS7 is a monofunctional pyran synthase (PS) domain that was predicted to catalyse ring formation from an α,β-unsaturated intermediate in the biosynthesis of the PKS–NRPS hybrid product pederin (**24**) [16–17]. The recombinant, isolated domain transformed both enantiomers of the structurally simplified tetraketidic precursor surrogate **22** into cyclised products *anti*-**23** and *syn*-**23** (Scheme 3a) [14]. The in vitro reaction with PedPS7 proceeds with moderate stereoselectivity irrespective of the configuration of the substrate at C7.

PS domains are common in *trans*-AT PKS clusters and participate in the biosynthesis of such important compounds as bryostatin and sorangicin. They are related to DH domains on the amino acid sequence level, but show a significant mutation of a DH-characteristic aspartic acid to a histidine or an asparagine residue in their active site. This exchange avoids the dehydration reaction and might facilitate the activation of the hydroxy group for nucleophilic attack on the Michael system by proton abstraction. PS domains also form a distinct phylogenetic clade compared to DH domains. Within a module, PS domains are usually located adjacent to DH domains and act on their transiently formed dehydration product [14].

The arrangement is somewhat different in the case of AmbDH3 from ambruticin biosynthesis (Scheme 4) [15]. This bifunctional domain catalyses both steps, dehydration of a 3-hydroxythioester intermediate **25** and subsequent cyclisation to a tetrahydropyran ring **27**. AmbDH3 is currently the only known case of a pyran-forming domain in a *cis*-AT PKS.

Hahn et al. showed that AmbDH3 catalyses dehydration of only the 2-D,3-D-configured precursor **25** to the *E*-configured olefin intermediate **26** and subsequent cyclisation to **27** (Scheme 4). The C6 epimers of compounds **25** and **26** were also accepted, but with much lower conversion. In both cases, the configuration at C2 in the cyclic product was exclusively D, highlighting the high stereoselectivity of the domain-catalysed reaction. The ambruticins contain a second hydropyran ring that is established by epoxide opening (see chapter 1.1.3).

**Scheme 5:**
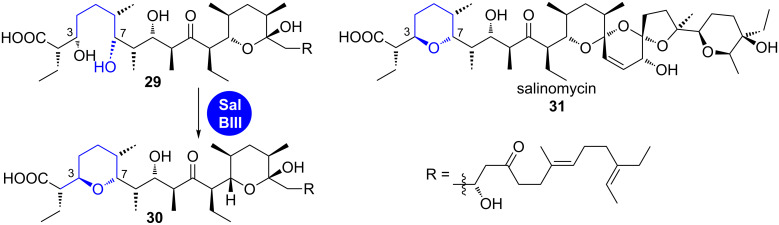
SalBIII catalyses dehydration of **29** and subsequent cyclisation to tetrahydropyran **30** [18].

A further enzyme with similar dehydratase–cyclase activity was recently discovered by Leadlay et al*.* in the biosynthesis of the polyether ionophore salinomycin (**31**, Scheme 5) [18]. SalBIII is a pyran-forming cyclase that was originally annotated as an epoxide hydrolase/cyclase.

The putative biosynthetic precursor **29** was isolated from a gene knockout strain and used in an in vitro assay. The recombinant enzyme converted this compound into the cyclised salinomycin precursor **30**. The proposed mechanism also proceeds via a dehydration–oxa-Michael addition cascade. A crystal structure revealed two crucial aspartic acid residues as candidates for the acid–base catalysis occurring in the active site [18].

**1.1.2 Processing of hemiacetals:** Reduction or alkylation of hemiacetals in the presence of Lewis acids is a common synthetic strategy for making pyrans and furans. Hemiacetals are also biosynthesis intermediates where they are transformed into individually functionalised heterocycles or acetals. In many cases, these hemiacetals are also appropriately activated to react further spontaneously. The involvement of individual enzymes in these reactions has only been shown in a few cases.

**Figure 1:**
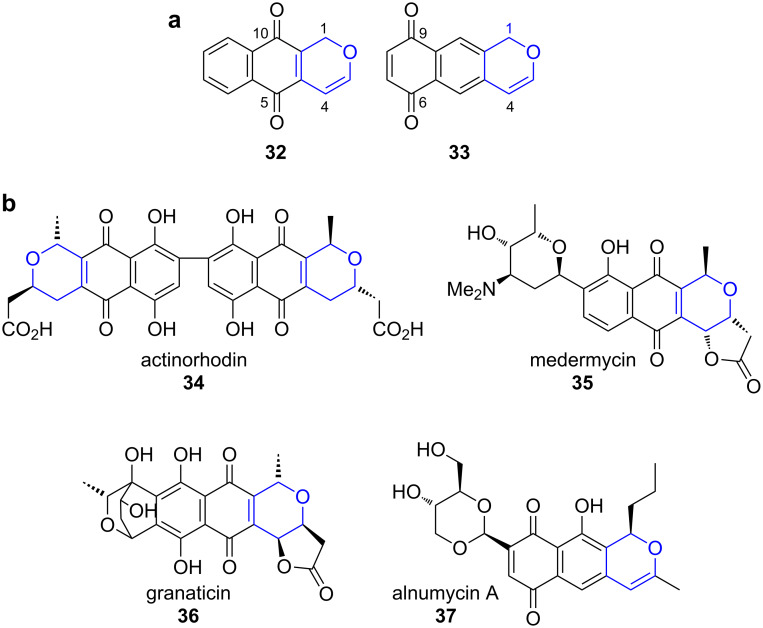
All pyranonaphtoquinones contain either the naphtha[2,3-*c*]pyran-5,10-dione (**32**) or the regioisomeric naphtha[2,3-*c*]pyran-6,9-dione (**33**) unit. Representative examples are actinorhodin (**34**), medermycin (**35**), granaticin (**36**) and alnumycin A (**37**) [21].

**Pyranonaphtoquinones.** Pyranonaphtoquinones are a subclass of bacterial and fungal polyketides with an aglycone core, which is built up of a naphthalenedione and an annelated pyran ring (Figure 1) [19–20].

Their biosynthesis can be divided into three parts: the assembly of the PKS carbon backbone by a type II PKS including formation of the carbocyclic aromatic core, post-PKS modifications leading to the installation of the oxygen heterocycle and third, its modification by diverse tailoring enzymes [21–22].

**Scheme 6:**
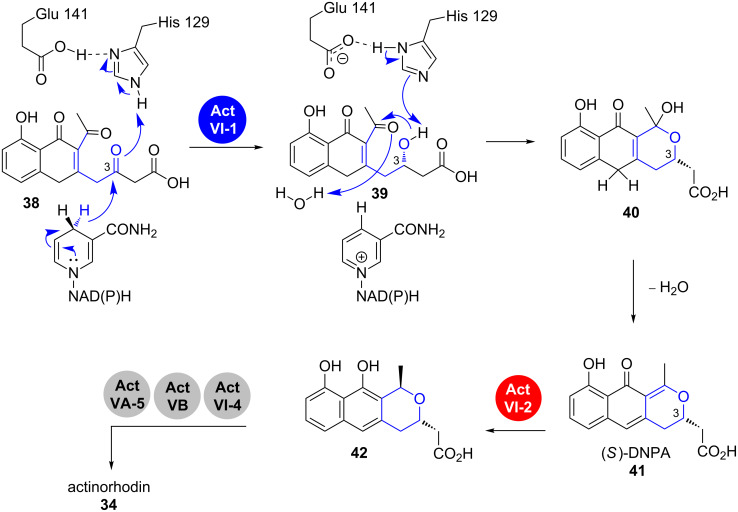
Pyran-ring formation in actinorhodin (**34**) biosynthesis. DNPA: 4-dihydro-9-hydroxy-1-methyl-10-oxo-3*H*-naphto[2,3-*c*]pyran-3-acetic acid. Modified from [27–29].

The actinorhodin (**34**) PKS is probably the best studied type II PKS and has been used as a model system for understanding basic features of such iterative bacterial systems. The respective biosynthetic gene cluster had already been cloned in 1984 and the genes were sequenced in 1992 (Scheme 6) [23–24].

As for most pyranonaphtoquinones, seven rounds of chain extensions followed by controlled cyclisation yield the reactive intermediate **38** after release from the PKS [25–26]. The intermediate is prepared for the action of ActVI-1, which is annotated as a 3-hydroxyacyl-coenzyme A (CoA) dehydrogenase (3HAD). Enzymes of this family catalyse the dehydration of L-3-hydroxyacyl-CoA during β-oxidation of fatty acids.

In this case, it acts as a ketoreductase that installs the secondary hydroxy group in **39**. The catalytic mechanism has been proposed using a homologous 3HAD from the human heart as a model and was verified by mutagenesis and kinetic studies. In the active site, Glu141 and His129 activate the C3 keto group by protonation. The pro-*S* hydride of the reduced nicotinamide adenine dinucleotide (phosphate) (NAD(P)H) is then transferred to the C3. The resulting hydroxy group participates in the formation of a cyclic hemiacetal that subsequently undergoes vinylogous dehydration to yield (*S*)-4-dihydro-9-hydroxy-1-methyl-10-oxo-3*H*-naphto[2,3-*c*]pyran-3-acetic acid (**41**, (*S*)-DNPA) [27]. Whether the enzyme actively participates in the post-reduction steps is still in debate. It was proposed that dehydration takes place while the substrate is still bound in the active site, but it is also known that this and the analogous reaction in similar systems like medermycin (**35**) can occur spontaneously [27,30].

In vitro studies with recombinant ActVI-1 and synthetic substrate analogues showed a preference of the enzymes for a free acid substrate analogue over *N*-acetylcysteamine (SNAC)-bound substrates, suggesting that the polyketide is cleaved from the PKS prior to keto reduction [31]. The dehydration product **41** is reduced by Act VI-2 to the dihydropyran **42**, which undergoes tailoring to finally yield actinorhodin (**34**).

**Scheme 7:**
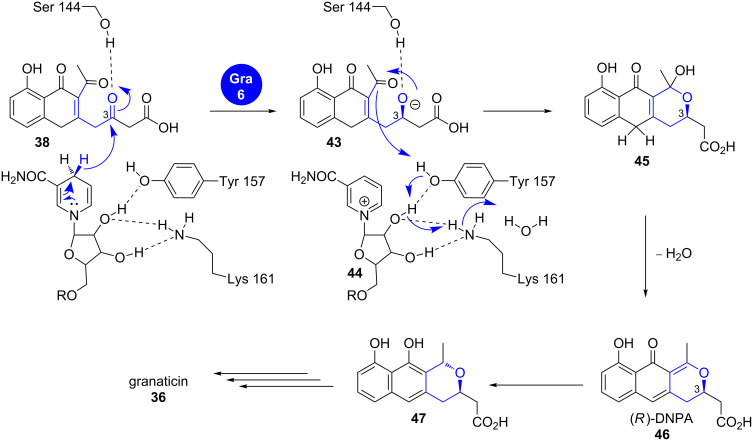
Pyran formation in granaticin (**36**) biosynthesis. DNPA: 4-dihydro-9-hydroxy-1-methyl-10-oxo-3*H*-naphto[2,3-*c*]pyran-3-acetic acid. Modified from [27].

In granaticin (**36**) biosynthesis, the pyran-forming enzyme Gra-6 belongs to the short chain dehydrogenase/reductase (SDR) family and shows the highly conserved catalytic triad of Ser-Tyr-Lys (Scheme 7).

It was suggested that the Ser144 hydrogen bond to the C3 keto group in **38** is essential for stereocontrol, while Tyr157 and Lys161 participate in pre-orienting NADPH for transfer of its pro-*S* proton [27,32]. The resulting secondary alcohol **43** is processed similar to its enantiomer **39** in actinorhodin biosynthesis to give (*R*)-DNPA (**46**) and finally graniticin (**36**) after tailoring.

**Scheme 8:**
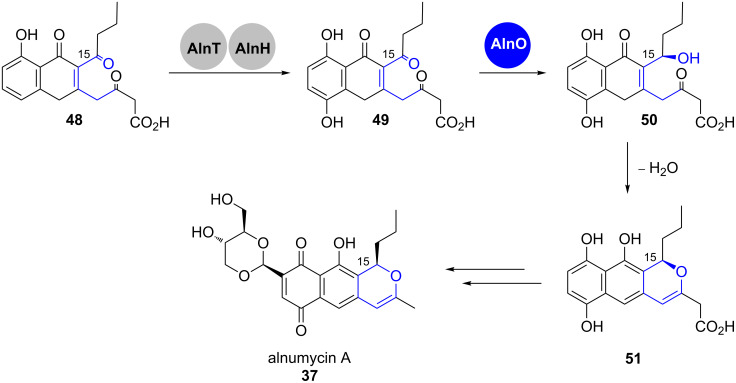
Pyran formation in alnumycin (**37**) biosynthesis. Adapted from [21].

It has been proposed that already at the stage of the first post-PKS modifications, the alnumycin (**37**) pathway differs from the above mentioned routes (Scheme 8). Prior to pyran cyclisation, the lateral ring of precursor **48** is hydroxylated by the combined action of the two-component flavin-containing monooxygenase (FMO) AlnT and the flavin reductase AlnH [33].

No 3HAD homolog is present in the gene cluster that could catalyse a similar reaction as in the above mentioned examples. Instead, the oxidoreductase AlnO was proposed to catalyse the stereoselective reduction of the ketone at C15 in **49**. The pyran **51** would then be obtained by spontaneous or enzyme-supported hemiacetalisation followed by dehydration [34]. The tricyclic core unit is oxidised further and heavily decorated by tailoring enzymes, also involving an unusual rearrangement leading to the dioxane unit, whose carbon atoms originally derive from a sugar building block [34–36].

**1.1.3 Epoxide opening:** The nucleophilic opening of epoxides is probably the most abundant type of reaction leading to furans and pyrans. It, for example, plays an important role in the biosynthesis of ionophoric terrestrial and marine polyethers (see chapter 1.3). In this chapter, we will focus on two examples in which one pyran ring is formed. Both characteristically deviate from the typical polyether-specific interplay between one epoxidase and one or a few epoxide hydrolases that collaboratively set up multiple oxygen heterocycles.

**Scheme 9:**
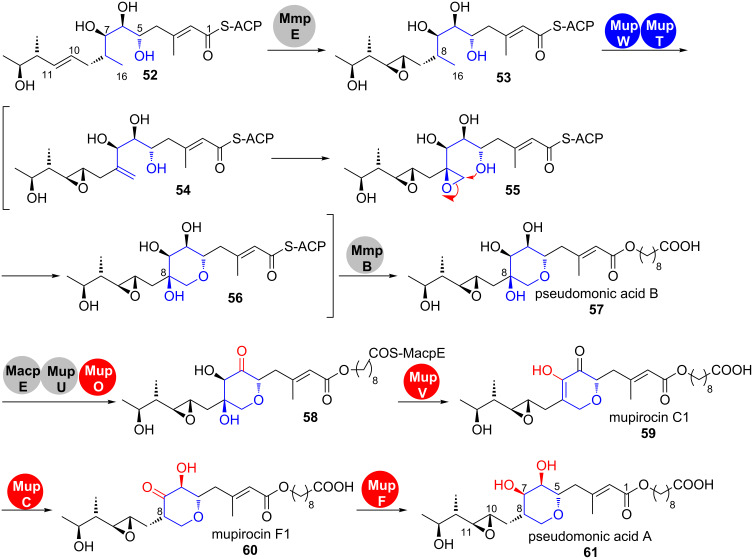
Biosynthesis of pseudomonic acid A (**61**). The pyran ring is initially formed in **57** after dehydrogenation, epoxidation and ring opening by the ferredoxin dioxygenase MupT and the dioxygenase MupW and then formally deoxygenated [46].

**Pseudomonic acid A.** Mupirocin is a clinically important antibiotic against Gram-positive bacteria, which consists of a mixture of pseudomonic acids from *Pseudomonas fluorescens* NCIMB 10586 with pseudomonic acid A (**61**) being the main compound (Scheme 9) [37–44]. It belongs to the group of *trans*-AT-PKS products and the gene cluster harbours genes that code for a β-hydroxymethylglutaryl-CoA synthase (HCS) cassette (*mupG*, *mupH*, *mupJ*, *mupK* and *macpC*) and an iteratively acting type I fatty acid synthase (FAS) (*mmpB*).

During the biosynthesis of the pseudomonic acids, the initially formed PKS product **52** undergoes a complex tailoring pathway (Scheme 9) [45]. A remarkable feature is the tightly regulated steps that lead to the formation and decoration of the pyran-ring-containing region between C5 and C11 in **61** [46–49]. This has been studied by a series of fermentation and gene deletion–intermediate isolation experiments.

The process starts by oxidoreductase domain MmpE-catalysed epoxidation of the double bond between C10 and C11. Olefin **53** is thus a branching point from which two series of analogous C10–C11 epoxides (**53**–**61**) and C10–C11 (not shown) olefins arise (Scheme 9). The fact that the wild-type titers of the respective olefins are much lower than the analogous epoxides **53**–**61** suggests that epoxidation has a strong influence on the performance of the downstream enzymes.

The dioxygenase MupW together with its associated ferredoxin dioxygenase MupT then catalyse dehydrogenation and epoxidation on C8 and C16 of **53**. Whether the pyran-ring closure is also mediated by an enzymatic activity or if this reaction is a spontaneous process could not be clarified yet and may be subject for in vitro studies with the purified enzymes.

The net-deoxygenation on C8 of pseudomonic acid B (**57**) is obtained by a multistep process (Scheme 9). After elongation by the iterative type I fatty acid synthase MmpB, redox transformations and a dehydration on the MacpE-bound substrate **58** finally lead to pseudomonic acid A (**61**) with a 3,4-dihydroxy-2,5-disubstituted pyran ring. The reason for the elaborate oxidation–reduction on the C6 and C7 hydroxy groups during this biosynthetic endgame remains enigmatic [46].

**Scheme 10:**
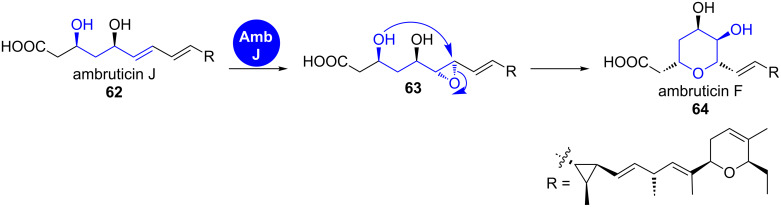
Epoxidation–cyclisation leads to the formation of the tetrahydropyran ring in the western part of the ambruticins [50].

**Ambruticin.** Another example in which a single epoxide opening event leads to the installation of an individual ring is the formation of the western tetrahydropyran ring in the biosynthesis of the ambruticins (Scheme 10) [50].

Their gene cluster contains a single epoxidase gene but no epoxide hydrolase, suggesting that the epoxidase is either multifunctional or that epoxide opening occurs spontaneously. The latter hypothesis is supported by the fact that allylic epoxides have been shown in synthetic experiments to be much more susceptible to nucleophilic attack than the respective 3,4-saturated analogues and that 6-*endo*-tet attack can override the 5-*exo*-tet cyclisation, which is favoured according to Baldwin’s rules [51–52].

#### 1.2 Furans

**1.2.1 oxa-Michael addition:** Similar to the PS domains described in chapter 1.1.1, furan rings can also be biosynthesised via oxa-Michael additions.

**Scheme 11:**
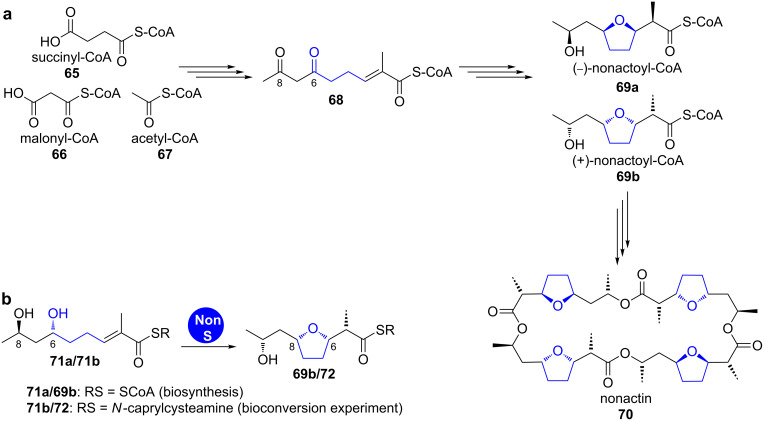
a) Nonactin (**70**) is formed from heterodimers of (−)(+)-dimeric nonactic acid and (+)(−)-dimeric nonactic acid. b) The product of the *nonS* gene catalyses the cyclisation of (6*R*,8*R*,*E*)-6,8-dihydroxy-2-methylnon-2-enoic acid thioester (**71a** and **71 b**) to (+)-nonactic acid thioester (**69b**/**72**) [53,56,58].

**Nonactin.** Nonactin (**70**) is the smallest homolog of the macrotetrolides, a family of cyclic polyethers that commonly have activity as ionophore antibiotics (Scheme 11a). It is produced by *Streptomyces griseus* subsp. *griseus* ETH A7796 as well as by *Streptomyces fulvissimus* and consists of four nonactic acid units, which are assembled in a head-to-tail fashion giving a *C*_2_-symmetric (−)-(+)-(−)-(+) macrocycle [53].

Nonactin (**70**) biosynthesis has been extensively studied and shows multiple unusual features. Genes of an ACP-less, non-iteratively acting type II PKS are involved in the formation of the nonactoyl-CoA (**69a** and **69b**) backbone. The biosynthesis starts from succinyl-CoA (**65**) and malonyl-CoA (**66**), which are condensed to a 3-oxothioester and further processed to the 4,6-dioxothioester **68** (Scheme 11a) [54]. This achiral intermediate is the precursor for two enantiospecific pathways [55].

After stereoselective reduction to the (6*S*,8*S*) or the (6*R*,8*R*, **71a**) enantiomer of (*E*)-6,8-dihydroxy-2-methylnon-2-enoyl-CoA, respectively, the nonactate synthase NonS catalyses stereospecific oxa-Michael addition [56–57]. This enzyme is proposed to convert both enantiomers, finally giving the nonactic acid monomers **69a** and **69b**.

Priestley et al. showed that the cell lysate of a recombinant *Streptomyces lividans* strain overexpressing the *nonS* gene was able to convert the *N*-caprylcysteamine thioester (**71b**) into the respective cyclic compound **72** (Scheme 11b) [56]. This result has been confirmed by in vivo experiments of Shen et al. [59]. NonS was annotated as an enoyl-CoA hydratase and shows a high degree of up to complete identity amino acid homology to mostly uncharacterised enzymes in several other clusters of *Streptomyces* strains.

**Figure 2:**
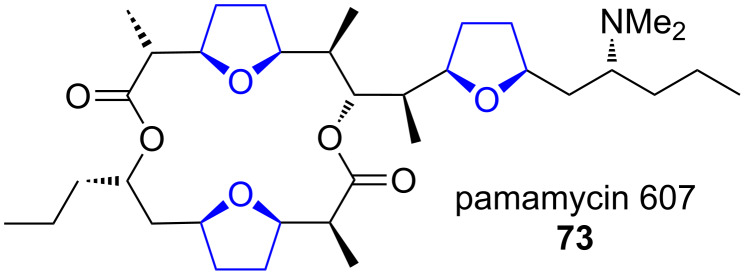
Pamamycins (**73**) are macrodiolide antibiotics containing three tetrahydrofuran moieties, which are all proposed to be formed by the NonS homolog PamS [60].

**Pamamycins.** Recently, the gene cluster of the macrodiolide antibiotic group of the pamamycins **73** was sequenced and the function of some of the genes studied (Figure 2) [60].

This cluster contains a NonS homolog, PamS, that was proposed to catalyse all three oxa-Michael additions that lead to tetrahydrofuran formation during biosynthesis. As the enzyme must act on biosynthetic intermediates of strongly varying size, this attributes a remarkably broad substrate tolerance to PamS. No detailed characterisation of PamS has been carried out yet.

**Oocydin.** Homologs of *trans*-AT-PKS-characteristic pyran synthase (PS) domains are also proposed to be involved in the biosynthesis of furan-containing compounds (see chapter 1.1.1).

**Scheme 12:**
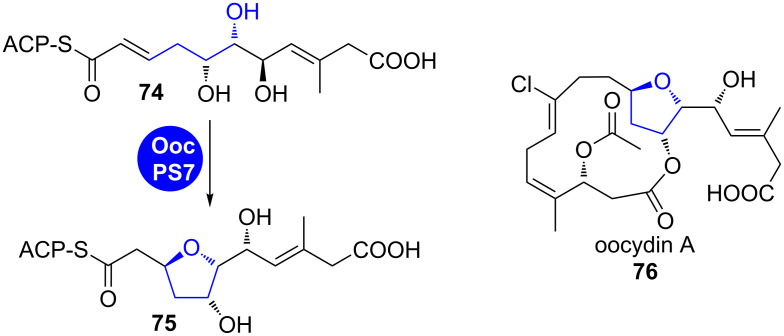
A PS domain homolog in oocydin A (**76**) biosynthesis is proposed to catalyse furan formation via an oxa-Michael addition [61].

In module 7 of the oocydin A (**76**)-PKS, a DH domain and a PS domain are present that were proposed to first dehydrate a 3-hydroxythioester intermediate and then cyclise the resulting enoyl intermediate **74**, similar to the reaction in sorangicin or pederin (**24**) biosynthesis (Scheme 12) [61].

**Scheme 13:**
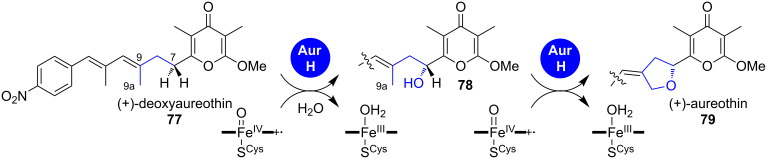
Mechanism of oxidation–furan cyclisation by AurH, which converts (+)-deoxyaureothin (**77**) into (+)-aureothin (**79**) [13].

**1.2.2 Oxidative cyclisation: (+)-Aureothin.** Furan rings can also be directly formed by oxidative cyclisation. The best studied example is the biosynthesis of (+)-aureothin (**79**), a reduced polyketide with potent antitumor, antifungal, antiparasitic, pesticidal and antitrypanosomal activities (Scheme 13).

In its biosynthesis, the furan-ring formation occurs on a late stage, catalysed in an unprecedented fashion by the cytochrome P450 oxidase AurH [13,62–67]. This enzyme accomplishes two consecutive CH activations at the positions 7 and 9a of the biosynthetic precursor deoxyaureothin (**77**), finally leading to oxidative cyclisation. The authors could reconstitute the enzymatic reaction in vitro and showed that stereospecific oxidation of **77** occurs first at the 7-position, which is followed by allylic oxidation at the 9a-position in **78** and cyclisation [13]. This reaction was exploited in the chemoenzymatic total synthesis of (+)-aureothin (**79**) [67–68].

The molecule also contains a pyran-4-one. Reminiscent of type II and type III-PKS, this results from elimination–tautomerisation of the 3,5-dioxothioester formed by the final two elongation steps of the aureothin-PKS.

**Scheme 14:**
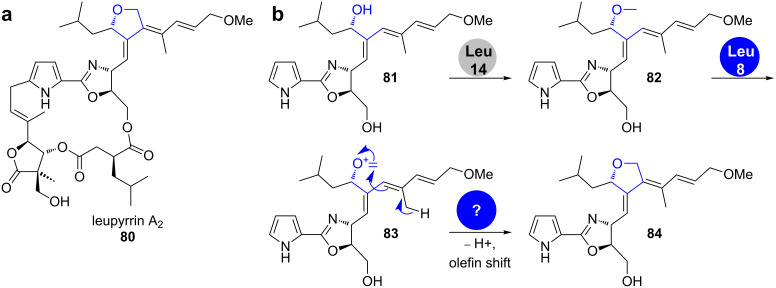
Leupyrrin A_2_ (**80**) and the proposed biosynthesis of its furylidene moiety [69–70].

**Leupyrrins.** The leupyrrins (leupyrrin A_2_ (**80**) is shown in Scheme 14) are remarkable hybrid natural products consisting of PKS, NRPS and isoprenoid-originating portions. They contain several heterocyclic elements, like a pyrrolidine, a furan-2-one, an oxazolidinone and particularly a 3,4-furylidene moiety in the polyketide part.

An analysis of the gene cluster as well as feeding experiments with isotope-labelled precursors led to a proposal for the formation of the furan ring [69–70]. The anticipated mechanism is reminiscent of the formation of a tetrahydropyridine ring by the berberine bridge enzyme in plant alkaloid biosynthesis. It starts with *S*-adenosyl-L-methionine (SAM)-dependent methylation of the secondary hydroxy group in **81** by the *O*-methyltransferase Leu14 (Scheme 14a) [71–74]. Oxidation of the methoxy group in **82** by the cluster-encoded dehydrogenase Leu8 is followed by a Prins-type cyclisation. No enzyme candidate for the cyclisation reaction to **84** could be identified in the cluster.

**Scheme 15:**
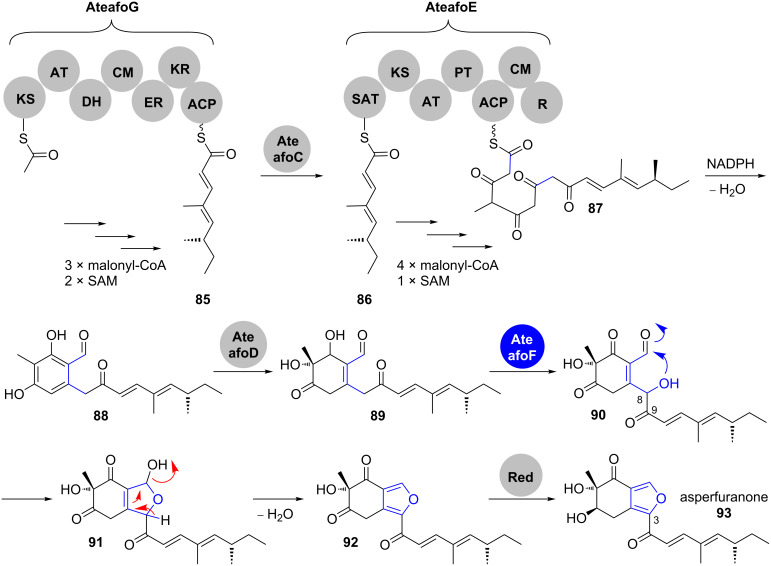
Asperfuranone (**93**) biosynthesis, adapted from [75].

**1.2.3 Processing of hemiacetals: Asperfuranone.** Asperfuranone (**93**) consists of a polyketide side chain, attached to the C3 of an oxidised isobenzofuran (Scheme 15). The respective biosynthetic cluster contains seven genes and has been identified by Wang and co-workers through a genome mining approach in *Aspergillus nidulans* [76]. Later on, the same group annotated a highly homologous gene cluster in *Aspergillus terreus* and elucidated the timing and mechanism of asperfuranone biosynthesis by step-wise heterologous expression of the individual genes in *A. nidulans* [77]. Thus, genes involved in asperfuranone biosynthesis have been renamed from “*afo*” to “*ateafo*”.

This bipartite azaphilone structure corresponds to its assembly by the highly reducing (HR)-PKS AteafoG, followed by a non-reducing (NR)-PKS AteafoE. The product of the HR-PKS AteafoG, tetraketide **85**, is transferred to the starter unit:ACP transacylase (SAT) domain of the NR-PKS AteafoE. After the elongation by four further ketide units, reductive PKS release and Knoevenagel condensation yield the benzaldehyde intermediate **88**. Oxidative dearomatisation of **88** catalysed by the salicylate monooxygenase AteafoD gives **89**, which is hydroxylated at C8 by the oxygenase AteafoF. The positioning of this newly formed hydroxy group forces the formation of a five-membered ring hemiacetal in **91**. Spontaneous dehydration installs the furan moiety and after keto reduction by an endogenous reductase, asperfuranone (**93**) is obtained.

**Figure 3:**
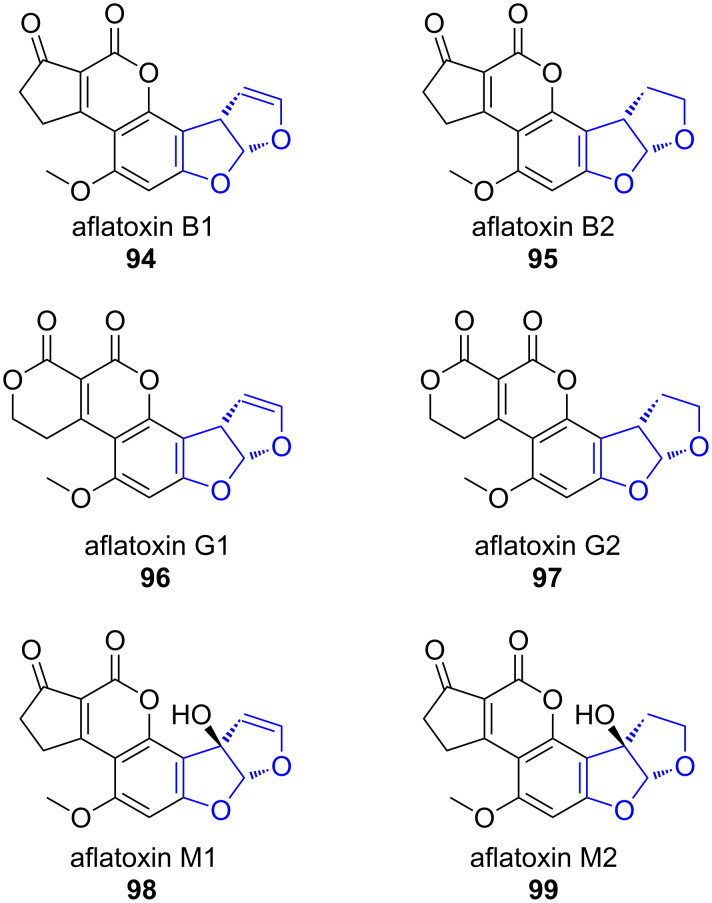
The four major aflatoxins produced by *Aspergilli* are the types B1, B2, G1 and G2 (**94**–**97**). In the digestive tract of animals, aflatoxins B1 and B2 (**94** and **95**) are oxidized to M1 and M2 (**98** and **99**), respectively [80–81].

**Aflatoxins.** Aflatoxins **94**–**99** are highly toxic carcinogens produced in several *Aspergillus* species (Figure 3). The respective pathway gene clusters have been identified and homologies between *Aspergillus* species were compared for example by the groups of Bennett and Ehrlich [78–79]. Structurally, aflatoxins belong to the group of furanocoumarins and consist of a pentacyclic system in which a benzobisfuran is annelated with a δ-lactone and a cyclopentanone or oxidation products of the latter.

Aflatoxin biosynthesis has been studied since the late 1960s and has attracted attention, because the polyketide undergoes a series of oxidative rearrangements, which drastically alter the molecular scaffold. Due to the complexity of these processes, we will focus on the steps directly associated with heterocycle formation [82–84].

**Scheme 16:**
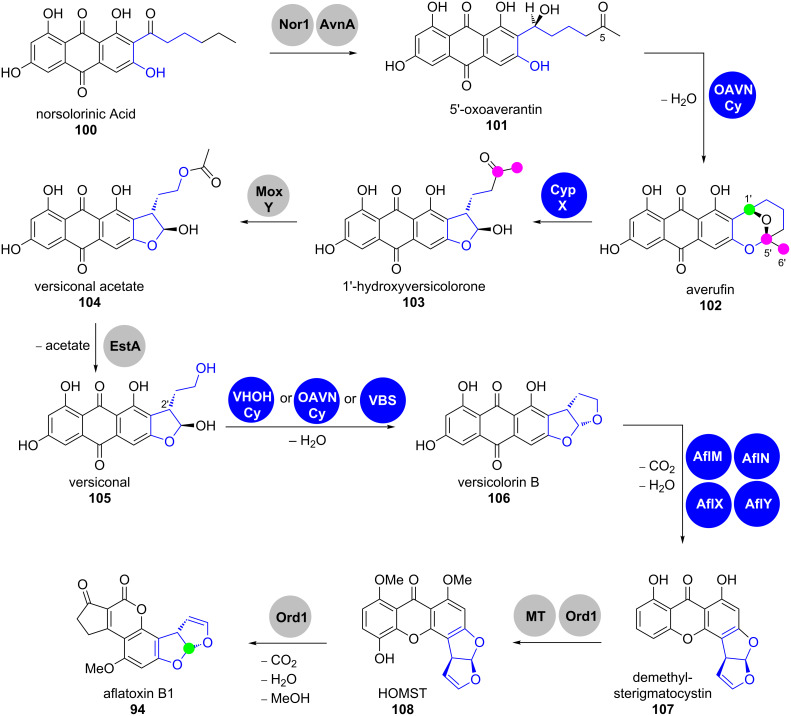
Overview on aflatoxin B1 (**94**) biosynthesis. HOMST = 11-hydroxy-*O*-methylsterigmatocystin [78–79,82–106].

Aflatoxin B1 (**94**) is considered as the most toxic aflatoxin. It is derived in multiple enzymatic conversions from norsolorinic acid anthrone **100**, which is produced by the norsolorinic acid synthase (NorS) (Scheme 16) [83,85]. NorS is a complex of a NR-PKS PksA and a pair of yeast-like fatty acid synthases HexA/HexB, which provide an unusual hexanoyl-CoA starter unit [86]. Norsolorinic acid (**100**) undergoes three oxidative rearrangements towards aflatoxin B1 (**94**): The first rearrangement sets up the benzobisfuran motif in **106**, the second rearranges the anthraquinone in **106** to the xanthone in **107** and the third is an oxidative ring contraction towards the cyclopentanone in **94** (Scheme 16).

After several enzymatic post-PKS modifications, the oxoaverantin (OAVN) cyclase transforms 5’-oxoaverantin (**101**) into averufin (**102**) by intramolecular acetal formation [87]. To date, it is not clear, how exactly the OAVN cyclase participates in this process [88]. Interestingly, the OAVN cyclase operates cofactor-free, although it contains a NAD(P)^+^-binding Rossman fold. Furthermore, this enzyme is also capable of catalysing the later conversion of versiconal (**105**) to versicolorin B (**106**) [88].

Averufin (**102**) is the starting point for the first oxidative rearrangement. Feeding experiments with isotope-labelled averufins (**102**) showed that their C5’ and C6’-carbons (pink) are excised on the way to aflatoxin B1 (**94**) and that the oxidation state of C1’ (green) changes from that of an alcohol to an aldehyde, implying that the rearrangement must be oxidative [82,89,91–92,107].

The biosynthetic mechanims of the conversion of averufin (**102**) into 1’-hydroxyversicolorone (**103**) has been the subject of intensive studies. Gene disruption experiments in the aflatoxigenic strain *A. parasiticus* NRRL 2999 revealed that this step is in fact catalysed by the cytochrome P450 enzyme AVR monooxygenase via an undeciphered mechanism (encoded by the gene *cypX*, see Scheme 16) [93]. The same study also revealed the participation of the FMO MoxY in a Baeyer–Villiger oxidation, which yields versiconal acetate (**104**) [93–94]. This is then hydrolysed by a cytosolic esterase (putatively also coded in the aflatoxin gene cluster as *estA*) to versiconal (**105**) [95]. The bisfuran moiety of versicolorin B (**106**), which is crucial for the mutagenic DNA binding, is then set up stereospecifically by the versiconal cyclase, which accepts both enantiomers (2’*R* and 2’*S*) of versiconal (**105**) [96,108]. Heterologous expression and characterisation by Townsend and co-workers revealed that the versicolorin B synthase (VBS) does not require any cofactors, in spite of its flavin adenine dinucleotide (FAD) binding site [98–99].

The reaction mechanisms and biosynthetic enzymes involved in the rearrangement of versicolorin B (**106**) to demethylsterigmatocystin (**107**) have also been discussed controversely. Up to four genes (*aflM*, *aflN*, *aflX* and *aflY*) have been implied in biosynthetic studies to code for enzymes that are participating in this complex conversion [100]. Henry and Townsend suggested an oxidation–reduction–oxidation sequence mediated by putative NADPH-dependent oxidoreductase AflM and cytochrome P450 enzyme AflN [101]. Gene disruption experiments by Cary et al. have shown that the NADH-dependent oxidoreductase AflX also takes part in the conversion [102]. Furthermore, the putative Baeyer–Villiger oxidase AflY was shown to be essential for demethylsterigmatocystin (**107**) formation and has been rationalised to form an intermediate lactone that is decarboxylated towards the xanthone [103]. Studies with recombinant AflM and a lack of isolatable intermediates however made it clear that the order of steps in demethylsterigmatocystin (**107**) formation needs to be carefully re-evaluated [100].

Methylation by an *O*-methyltransferase and subsequent oxidation by the cytochrome P450 monooxygenase Ord1 yields HOMST (**108**), which is the starting point for the final rearrangement towards aflatoxin B1 (**94**) [104–105]. Consequently, the Ord1 enzyme alone catalyses the final steps towards aflatoxin B1 (**94**) [106].

**1.2.4 Epoxide opening:** See chapter 1.1.3.

#### 1.3 Polycyclic systems

**Scheme 17:**
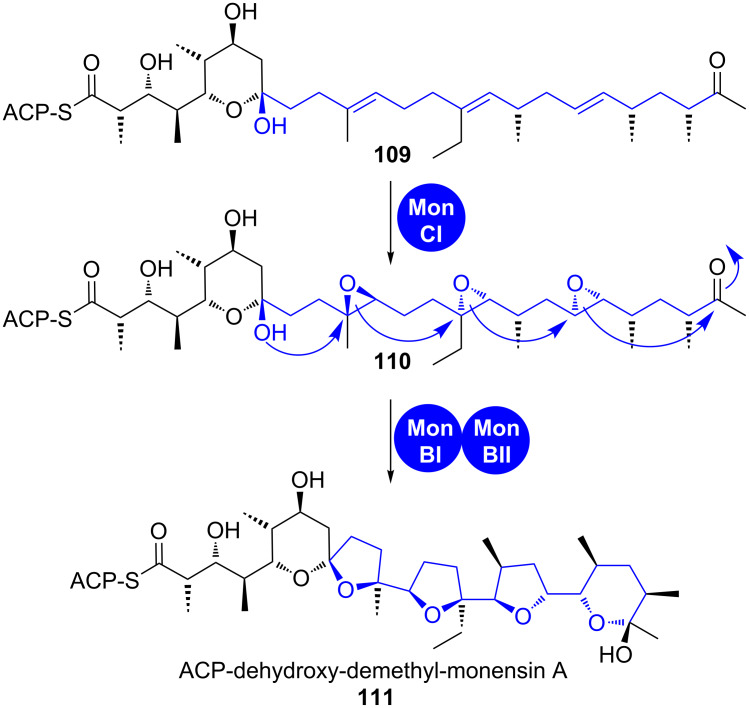
A zipper mechanism leads to the formation of oxygen heterocycles in monensin biosynthesis [109–111].

During the biosynthesis of ionophoric terrestrial and marine polyethers, polyolefinic PKS products are first polyepoxidised and these epoxides are then opened in a so-called zipper mechanism that installs furan and/or pyran rings as well as cyclic acetals, if carbonyl groups are involved (shown for monensin in Scheme 17) [109–111]. While polyepoxidation is usually effected by only one epoxidase, one or more epoxide hydrolases mediate regioselective epoxide opening and following controlled cyclisation.

As the topic polyether biosynthesis is highly complex and a detailed discussion of further examples would go beyond the scope of this article, we would like to refer the reader to appropriate review literature [4,109].

**Scheme 18:**
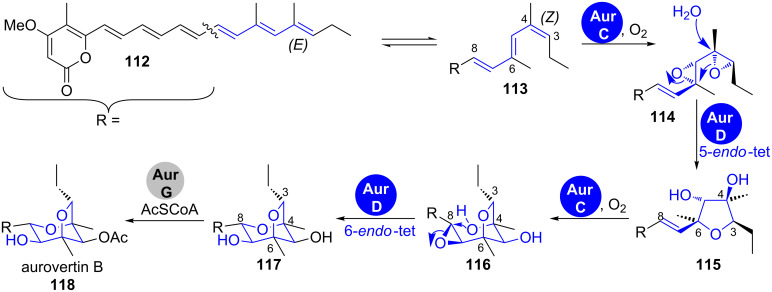
Formation of the 2,6-dioxabicyclo[3.2.1]octane (DBO) ring system in aurovertin B (**118**) biosynthesis [116].

**Aurovertin B.** The 2,6-dioxabicyclo[3.2.1]octane (DBO) ring system is present in several plant and microbial natural products, like decurrenside A from the goldenrod plant, sorangicin A from myxobacteria as well as the marine toxin palytoxin from zoanthids [112–114]. It was proposed that a complex epoxide opening cascade is involved in its formation (Scheme 18) [115].

Tang et al. were recently able to show that the interplay of one FMO and one epoxide hydrolase in the biosynthesis of the fungal polyketide aurovertin B (**118**) is sufficient to form this complex structural motif starting from a polyene-α-pyrone precursor (Scheme 18) [116]. The diene between C3 and C6 in **113** is first expoxidised by the FMO AurC. The epoxide hydrolase AurD then regioselectively hydrolyses the epoxide at the C4 position of **114** and initiates a cascade that leads to the formation of the dihydroxyfuran intermediate **115**. AurC becomes active for a second time and epoxidises the C7–C8 double bond, which is then attacked by the *syn*-positioned hydroxy group on C4 to give the pyran ring. This terminal 6-*endo*-tet cyclisation is likewise facilitated by AurD, overriding the 5-*exo*-tet pathway that should be favoured in spontaneous reaction according to Baldwin’s rules. Density functional theory calculations suggested that this reaction pathway is favoured, if the hydroxyepoxide **116** is simultaneously activated by acidic and basic residues [116].

#### 1.4 Oxetans

Oxetans are present in several isoprenoid natural products with important biological activity, like the anticancer drug paclitaxel or merrilactone A [117–122]. However, to the best of our knowledge, no information on the biosynthesis of polyketides containing this structural motif is known yet.

#### 1.5 Epoxides

**Figure 4:**
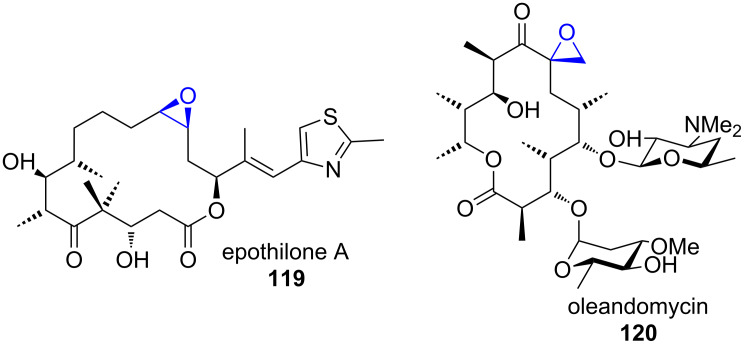
Structures of the epoxide-containing polyketides epothilone A (119) and oleandomycin (**120**) [123–125].

Epoxides are frequently occurring structural motifs in natural products and are often sites of covalent interaction with target proteins. Prominent compounds that contain epoxides are for example epothilone A (**119**) and oleandomycin (**120**) (Figure 4) [123–125].

Epoxides result from oxidation of olefins by oxidoreductases, mostly cytochrome P450 monooxygenases or FMOs. Alternative mechanisms, such as reactions between carbenes and carbonyls (analogous to the synthetic Corey–Chaykovsky epoxidation) are not known in biosynthetic pathways. Epoxides are abundant as biosynthetic intermediates that are further processed in downstream processes. Examples are discussed in the respective chapters 1.1.3 and 1.2.4.

#### 1.6 Pyranones

**Scheme 19:**
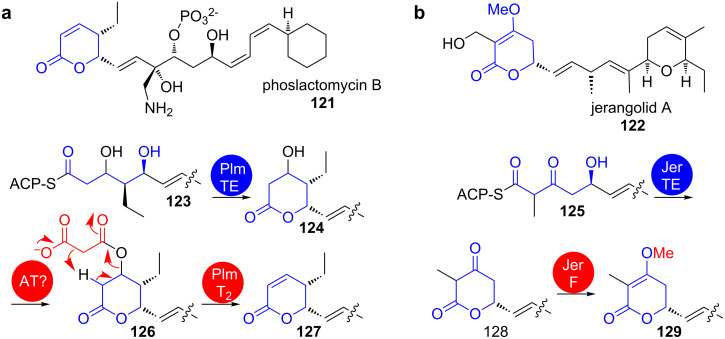
Structures of phoslactomycin B (**121**) (a) and jerangolid A (**122**) (b). The heterocycle-forming steps in their biosynthesis are shown on the bottom [50,127].

**1.6.1 TE-catalysed lactonisation:** Most pyranones occurring in polyketides are pyran-2-ones that result from the attack of 5-hydroxy groups or enols on the thioester of a PKS-bound intermediate. For a general introduction into this reaction in type II and type III PKS, we would like to refer the reader to the review article from Schäberle et al. published in this issue [126]. In type I PKS, cyclisation to pyran-2-ones usually occurs TE-catalysed after full assembly of the PKS product and is often followed by tailoring steps. Examples are the biosynthesis of jerangolid A (**122**) and of phoslactomycin B (**121**) (Scheme 19).

In phoslactomycin B (**121**) biosynthesis, the 4-hydroxytetrahydro-2*H*-pyran-2-one **124** is formally dehydrated by consecutive malonylation–elimination to finally give a 5,6-dihydro-2*H*-pyran-2-one **127** [127]. The tailoring enzyme PlmT_2_ was proposed to catalyse the decarboxylative elimination of malonoyl halfester **126**. It is not clear, whether the initial malonylation was catalysed by an AT domain or another enzyme in the cluster. Similar chemistry occurs during the biosynthesis of related compounds like fostriecin and leptomycin [128–129].

In jerangolid A biosynthesis, the dihydro-2*H*-pyran-2,4(3*H*)-dione **128** is transformed into a 4-methoxy-5,6-dihydro-2*H*-pyran-2-one **129** by action of the *O*-methyltransferase JerF [50].

**1.6.2 Michael addition–lactonisation:** A novel mechanism for the integration of pyran-2-ones into polyketide backbones has recently been discovered.

**Scheme 20:**
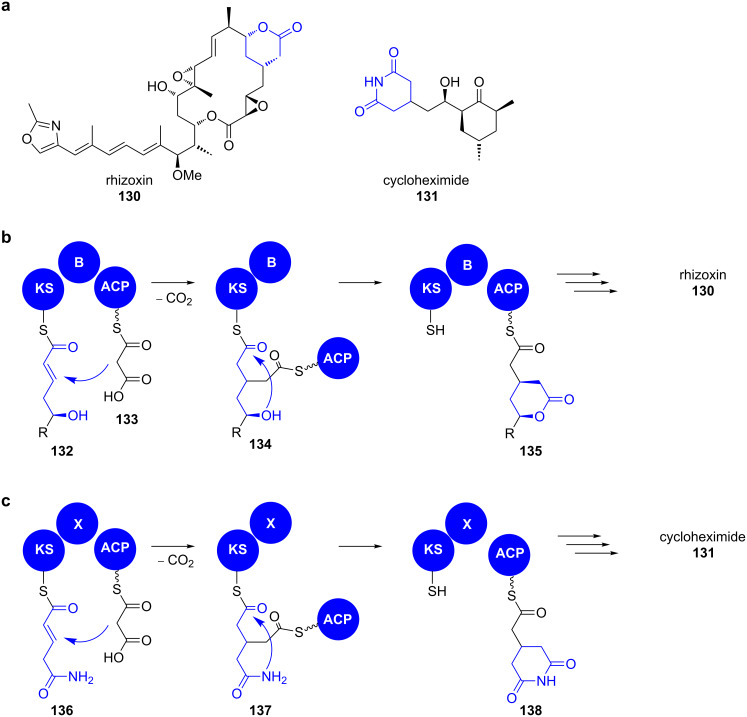
a) Structures of rhizoxin (**130**) and cycloheximide (**131**). Model for the formation of δ-lactones (b) or glutarimides (c), respectively. Adapted from [133].

**Rhizoxin.** In 2013, Hertweck and co-workers provided detailed insight into the unprecedented enzyme catalysis involved in the formation of 4-substituted δ-lactones and the structurally closely related glutarimides, respectively (Scheme 20) [130].

The assembly of both moieties includes a β-branching event of the polyketide carbon backbone that is mechanistically different from that occurring during isoprenoid biosynthesis. The designated branching modules of lactone and glutarimide-producing PKS show similar designs: a branching domain (B or X), which is flanked by a KS and an ACP domain (Scheme 20b and c).

In vitro reconstitution experiments with the branching module of the macrolide rhizoxin (**130**) (*rhi*PKS) and synthetic SNAC-thioesters revealed that the chain branch originates from a *syn*-selective Michael addition of an ACP-bound malonate unit **133** to a KS-bound α,β-unsaturated thioester **132** (Scheme 20b) [130]. This results in an intermediate **134** in which the ACP and the KS domain are covalently linked by the branched polyketide. Subsequent nucleophilic attack of the δ-hydroxy group on the thioester then yields the ACP-bound δ-lactone **135** and the polyketide chain can be passed downstream on the assembly line.

When testing the substrate scope of the rhizoxin (**130**) branching module, C3-substituted as well as amino and carboxamide nucleophiles in lieu of a hydroxy group in **132** were accepted, yielding δ-lactam and glutarimide moieties, respectively [131–132]. When the B-domain of the *rhi*PKS was exchanged with an X-domain of glutarimide-producing PKS from the 9-methylstreptimidone PKS of *S. himastatinicus*, both, glutarimides and lactones were obtained from respective substrate conversions. Thus, the domains can be seen as functionally equivalent [133]. Supported by kinetic analyses and mutational studies it was shown that B-domains, neither have an influence on the substrate selectivity nor on the turnover and furthermore do not catalytically take part in the branching or heterocyclisation event. Solely their double-hotdog fold is structurally essential for the branching module. Consequently, the B-domain has even been mimicked with a dehydratase domain that bears the same folding motif. It is thus most interesting that the branching KS domain alone mediates the entire catalytic sequence and represents a unique family of ligase-cyclase.

**Scheme 21:**
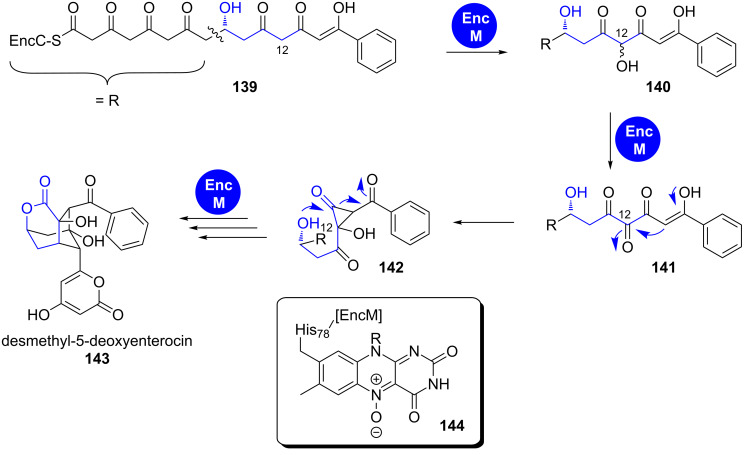
EncM catalyses a dual oxidation sequence and following processing of the highly reactive intermediate **141** in enterocin biosynthesis. EncC is an ACP. Adapted from [137].

**1.6.3 Favorskii rearrangement: Enterocin.** Another mechanism applies for the δ-lactone embedded in the tricyclic, caged core of the bacteriostatic agent enterocin that is produced in *Streptomyces* species [134–135]. The respective biosynthetic pathway has been fully reconstituted in an in vitro one-pot reaction [136]. The flavoprotein EncM transforms the C12 methylene group of the octaketidic PKS type II product **139** in a two-step oxidation sequence using the unprecedented, enzyme-bound flavin-*N*^5^-oxide **144** (Scheme 21) [137].

The resulting ketone **141** undergoes a Favorskii rearrangement, finally leading to the formation of the δ-lactone moiety. EncM has also been rationalised to participate in the stereoselectivity of the subsequent aldol condensations and the final lactonisation yielding the pyran-2-one attached to the caged ring system [137].

#### 1.7 Furanones

**Figure 5:**
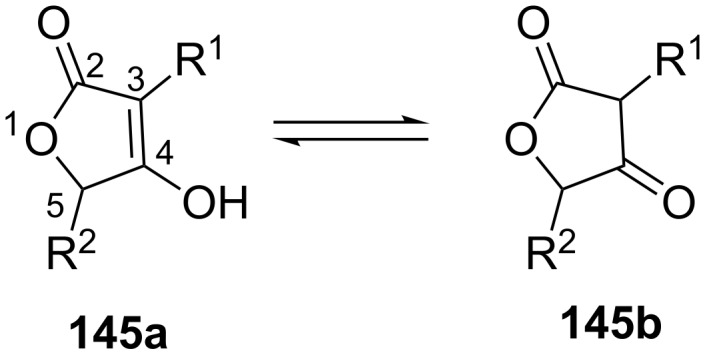
Mesomeric structures of tetronates [138–139].

**1.7.1 Acylation–Dieckmann condensation: Tetronates.** Tetronates (4-hydroxyfuran-2(5*H*)-ones, **145a/145b**) are an abundant type of heterocycles with a broad spectrum of biological activities (Figure 5) [138–139]. In polyketides, they mostly appear in form of 3-acyltetronates and it was proposed that this structural motif is able to mimic corresponding anions of acidic functional groups like phosphates, sulphates or carboxylates. In fact, tetronates often act by inhibiting enzymes that process the respective functional groups. Besides a common antibacterial activity, many tetronates also have further attractive bioactivities that triggered interest in their research [139–140].

**Figure 6:**
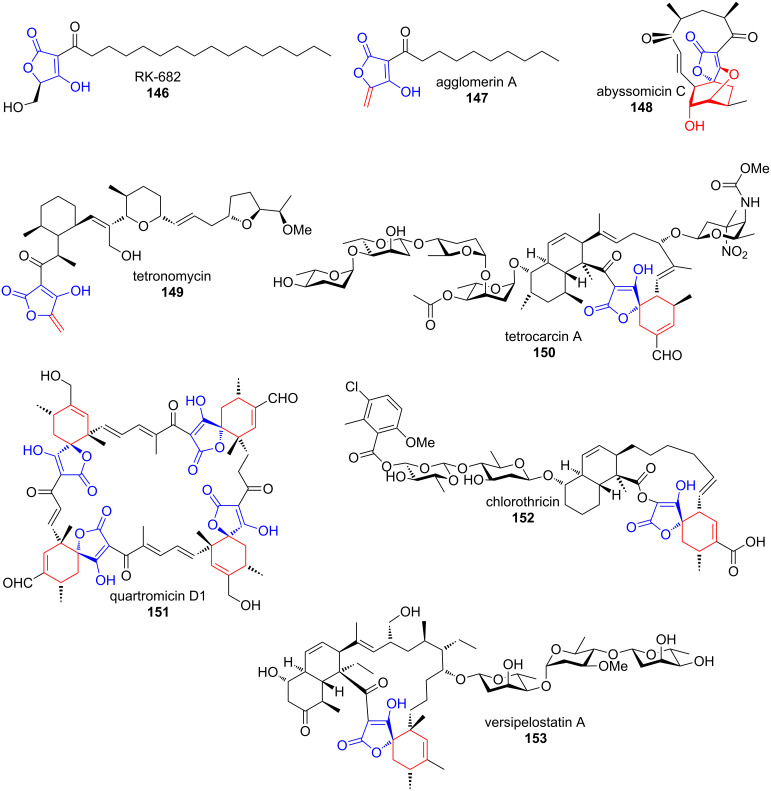
Structures of tetronates for which gene clusters have been sequenced. The tetronate moiety is shown in blue. All structural elements that derive from tailoring processes on the tetronate are shown in red. Kijanimicin is not shown [138–139].

Tetronates and their biosynthesis have recently been extensively reviewed and a classification based on structural characteristics was devised [138–139]. With the exception of a few terpenoids, most tetronates are of polyketide origin, either being completely biosynthesised by a PKS or by hijacking intermediates from fatty acid biosynthesis (Figure 6).

Although the larger body of tetronates is produced by *Actinobacteria*, they are abundant in organisms from different classes. Their origin often goes along with characteristic structural elements. Furylidene tetronates are for example exclusively produced by fungi and spirotetronates by *Actinobacteria* [139].

The biosynthetic studies that were launched since genetic information on more and more tetronates became available revealed that the formation and the decoration of the ring structure are straight forward and well-conserved among clusters and organisms.

**Scheme 22:**
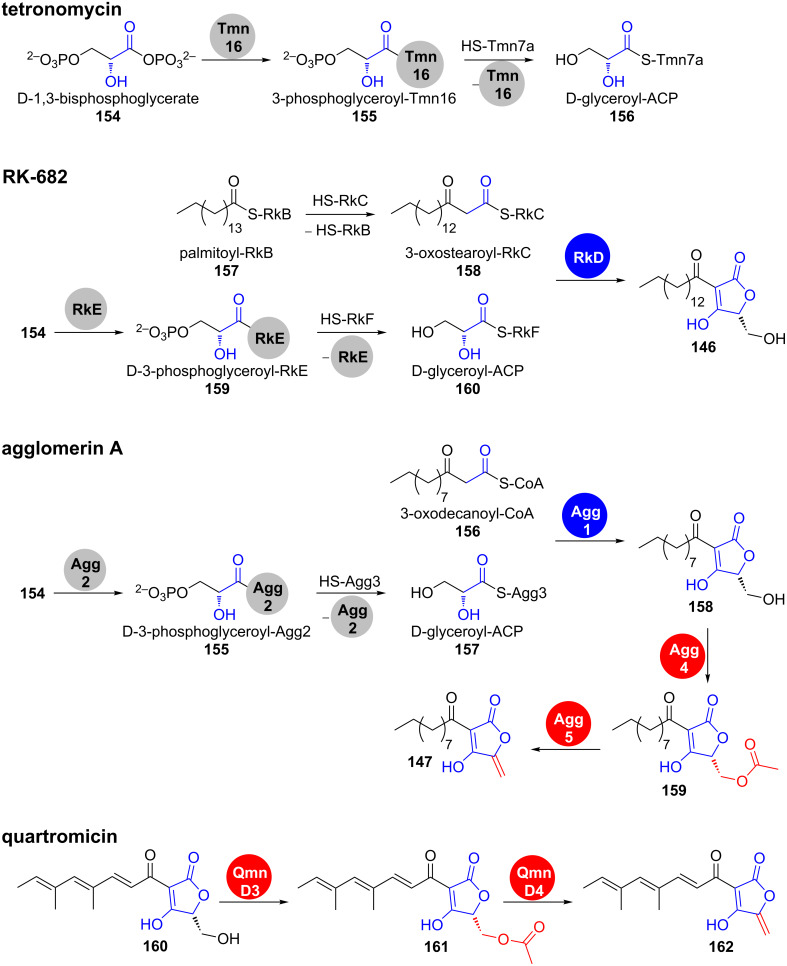
Conserved steps for formation and processing in several 3-acyl-tetronate biosynthetic pathways were confirmed by in vitro studies. Tmn7a, RkC, RkF and Agg3 are ACPs. Fragments, which are established by tetronate processing are shown in red [139,141–143].

**Tetronate cyclisation.** In all tetronate clusters sequenced until now, a conserved set of genes is present that codes for a glycerate-activating FkbH-like protein, an acyl carrier protein (ACP) that intermediately carries the glycerate unit and a FabH-like protein that condenses the ACP-bound glycerate with an ACP-bound β-ketothioester in an acylation–Dieckmann condensation reaction cascade (Scheme 22) [139]. This scenario has been confirmed by partial in vivo and in vitro reconstruction of tetronomycin (Tmn, **149**), RK-682 (**146**) and agglomerin A (**147**) biosynthesis [141–144]. It is however not clear, in which order the two sub-steps occur.

**Tetronate processing.** Spirotetronates like abyssomycin C (**148**), quartromycin D1 (**151**) or versipelostatin A (**153**) result from formal [4 + 2] cycloaddition reactions between *exo*-methylene groups and conjugated dienes. The required *exo*-methylene groups are installed by formal dehydration of 5-hydroxymethyltetronates.

Leadlay et al. have confirmed that the respective reaction in agglomerin A (**147**) biosynthesis proceeds as a two-step process [143]. An initial acyl transferase Agg4-catalysed acetylation of the primary hydroxy group in **158** is followed by dehydratase Agg5-catalysed acetic acid elimination, leading to olefin **147** (Scheme 22). This mechanism was confirmed by gene knockout and complementation experiments as well as by in vitro reconstitution using purified enzymes. Agg4 and Agg5 showed substrate tolerance and also accepted RK-682 as a substrate, thereby generating a novel agglomerin derivative. Similar genes are coded in all known clusters of spirotetronates. An analogous acetylation–elimination process was experimentally confirmed for quartromicin D1 (**151**) biosynthesis (Scheme 22) [145].

**Scheme 23:**
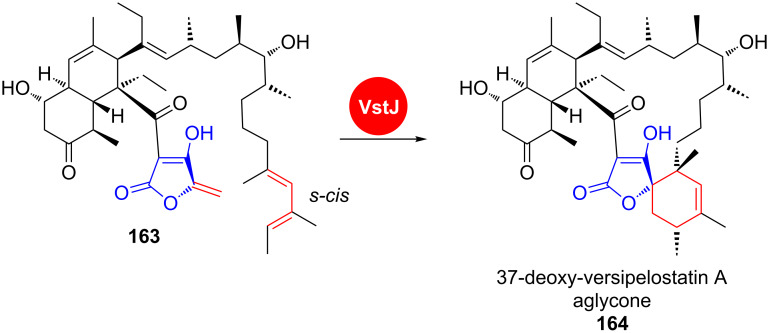
In versipelostatin A (**153**) biosynthesis, VstJ is a candidate enzyme for catalysing the [4 + 2] cycloaddition. VST: versipelostatin A [146].

VstJ has been identified as a probable candidate for the enzyme-catalysed [4 + 2] cycloaddition in versipelostatin A (**153**) biosynthesis by heterologous expression, gene knockout experiments and in vitro reaction with the purified enzyme (Scheme 23) [146].

Interestingly, homologs of *vstJ* are also present in the biosynthetic gene clusters of the spirotetronate-containing polyketides abissomycin C (**148**), tetrocarcin (**150**), quartromicin D1 (**151**), chlorothricin (**152**), lobophorin and kijanimicin. All these genes are remarkably small in size (*vstJ* for example codes for only 142 amino acids) and have no significant sequence similarity to other characterised proteins [146–147].

The homologous *qmnH* from quartromicin D1 (**151**) biosynthesis contains two tandem-*vstJ* sequences in agreement with the fact that four [4 + 2] cycloaddition events need to take place to assemble the four monomers into the highly symmetrical natural product [147].

**Scheme 24:**
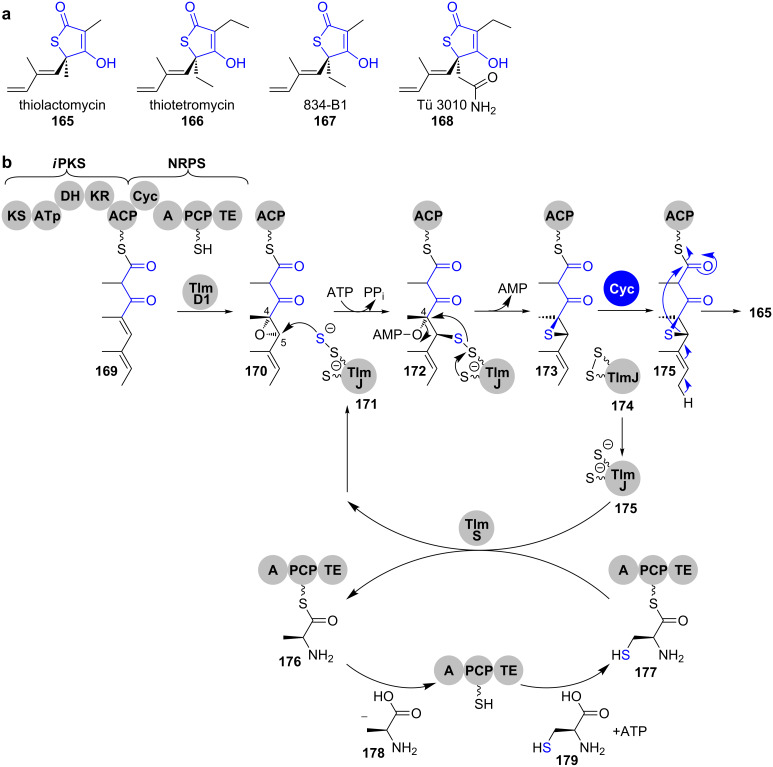
a) Structures of some thiotetronate antibiotics. b) Biosynthesis of thiolactomycin (**165**) as proposed by Leadlay and co-workers. The configuration of the stereocentres in the PKS intermediates is postulated based on the assumption that all reactions on the way to the structurally fully elucidated product **165** are occurring stereospecifically. Cyc: cyclase domain, A: adenylation domain, PCP: peptidyl carrier protein [148].

**Thiotetronates.** Recently, Leadlay et al. presented their findings on the biosynthesis of thiotetronate antibiotics (Scheme 24) [148]. These small heterocyclic compounds are produced by a range of actinomycetes and a deeper understanding of their biosynthesis was for a long time hampered by the inability to identify their biosynthetic genes.

Those were finally discovered by a comparative genomics approach in which the clusters of thiolactomycin (**165**), thiotetromycin (**166**), 834-B1 (**167**) and Tü 3010 (**168**) were sequenced and genetically manipulated (Scheme 24a). Gene knockout experiments and heterologous expression of the whole clusters as well as versions devoid of key genes revealed an unprecedented mechanism for heterocycle formation (shown for thiolactomycin (**165**) in Scheme 24b).

For thiolactomycin (**165**), an iteratively acting PKS module produces a tetraketide **169** that contains all backbone carbon atoms of the natural product and which is regioselectively epoxidised at the C4 and C5 carbons by the cytochrome P450 monooxygenase TlmD1 to give **170**. The peptidyl carrier protein (PCP) of the downstream NRPS module is loaded with an L-cysteine, which serves as a sulphur donor. From **177**, sulphur is transferred by the NifS-like cysteine desulphurase TlmS to the tRNA-specific and adenosine triphosphate (ATP)-dependent 2-thiouridylase TlmJ, which is thereby converted into its disulphide form **171**.

Disulphide attack on the C5 position of **170**, activation of the resulting secondary hydroxy group as the adenosine monophosphate (AMP) ester **172** and nucleophilic attack of the sulphur on the C4 position leads to thiirane **173** formation. The cyclase domain of the NRPS module would be responsible for double-bond shift and ring opening of the thiirane **173** with concomitant nucleophilic attack of the thiolate on the thioester, leading to thiolactone **165** formation along with the cleavage from the multienzyme.

As all key genes are also present in the clusters of the other thiotetronates **166**–**168**, it was postulated that this mechanism is general for the formation of this type of heterocycle.

**Scheme 25:**
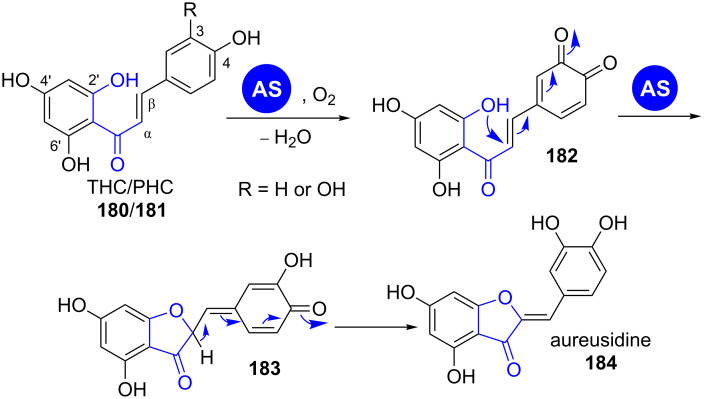
Aureusidine synthase (AS) catalyses phenolic oxidation and conjugate addition of chalcones leading to aureusidine (**184**). THC: 2’,4,4’,6’- tetrahydroxychalcone; PHC: 2’,3,4,4’,6’- pentahydroxychalcone [154].

**1.7.2 Oxidative cyclisation: Aurones.** Aurones are yellow coloured pigments of ornamental flowers that belong to the flavonoids. They are structurally closely related to chalcones, from which they differ by a central, annelated furan-3-one moiety instead of an acrylate unit (Scheme 25) [149]. Their biosynthesis proceeds from chalcones by an oxidation–conjugate addition cascade catalysed by plant phenol oxidases (PPOs) [150–151].

The PPO aureusidin synthase plays a central role in aurone biosynthesis in *Antirrhinum majus* [152–153]. It catalyses the oxidation of phenols **180** and *o*-catechols **181** to *o*-quinones **182** and concomitant conjugate addition of a phenolic hydroxy group, leading to the formation of the central furan-3-one unit [154]. This enzyme is flavin-dependent and acts under consumption of hydrogen peroxide.

It has been shown that the AS is substrate tolerant and accepts different hydroxylation patterns as well as glycosylations on the chalcone A and B rings [154]. However, the oxidative half-reaction only occurs with chalcones and not with other aryl substrates like L-tyrosine, 3,4-dihydroxy-L-phenylalanine (L-DOPA), 4-coumaric acid or caffeic acid.

**Scheme 26:**
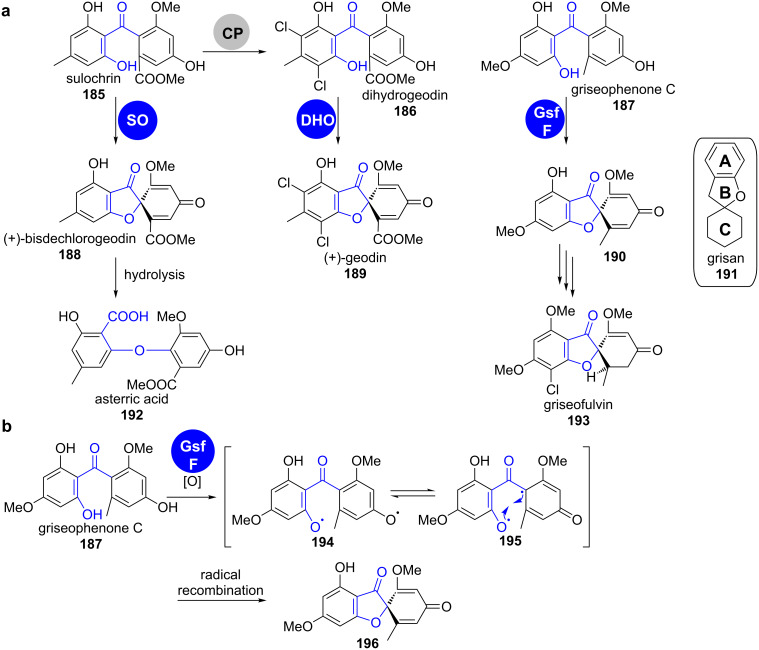
a) Oxidative cyclisation is a key step in the biosynthesis of spirobenzofuranes **189**, **192** and **193**. b) Mechanism of the proposed cytochrome P450-catalysed stereospecific radical coupling in the biosynthesis of griseofulvin (**193**). CP: chloroperoxidase; SO: sulochrin oxidase; DHO: dihydrogeodin oxidase [157–159].

**Grisanes.** Many fungal spirobenzofuranones contain the grisane (**191**) moiety as the central structural motif (Scheme 26a). The spiro linkage between the B and C rings is installed by oxidative phenol coupling starting from type II-PKS-derived anthraquinone precursors [155].

(+)-Geodin (**189**) was the first chlorinated compound isolated from fungi [156]. During its biosynthesis in *Aspergillus terreus*, the furan-3-one ring is closed by action of the multicopper blue protein dihydrogeodin oxidase on dihydrogeodin (**186**) (Scheme 26a) [157–158]. The direct precursor of dihydrogeodin (**186**) in this pathway, sulochrin (**185**), is also a substrate for a close homologue of dihydrogeodin oxidase (DHO). Sulochrin oxidase (SO) converts sulochrin (**185**) into (+)-bisdechlorogeodin (**188**), which then spontaneously hydrates to asterric acid (**192**), the end product of this pathway in *Penecillium frequentans* [158].

In 2010, the gene cluster of griseofulvin (**193**) was sequenced and analysed [159]. This cluster does not contain a multicopper blue protein, but instead the cytochrome P450 oxygenase GsfF. This enzyme has no other obvious role in biosynthesis and was proposed to catalyse the stereospecific oxidative radical-coupling reaction of griseophenone C (**187**, Scheme 26b).

#### 1.8 Oxetanones

**Scheme 27:**
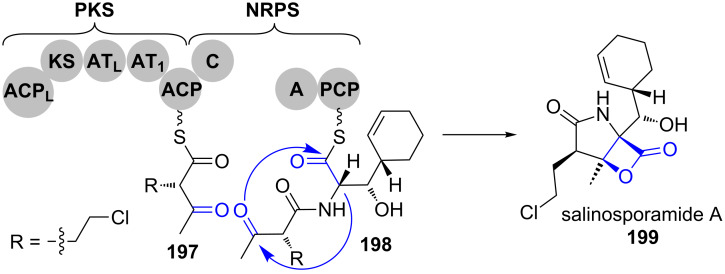
A bicyclisation mechanism forms a β-lactone and a pyrrolidinone and removes the precursor from the assembly line in salinosporamide A (**199**) biosynthesis [160–161].

**Salinosporamide.** Oxetanones are rare structures and highly reactive due to their ring strain. One of the most prominent examples is the proteasome inhibitor salinosporamide A (**199**) (Scheme 27) [160–161].

Based on gene cluster analysis, it was proposed that both heterocycles of this PKS–NRPS hybrid product, an oxetan-2-one and a pyrrolidin-2-one, are formed by a bicyclisation mechanism. Aldol addition of the amino acid α-position on the carbonyl gives the pyrrolidin-2-one. Concomitant attack of the intermediately formed hydroxylate on the thioester closes the β-lactone and releases salinosporamide A (**199**) from the assembly line.

**Scheme 28:**
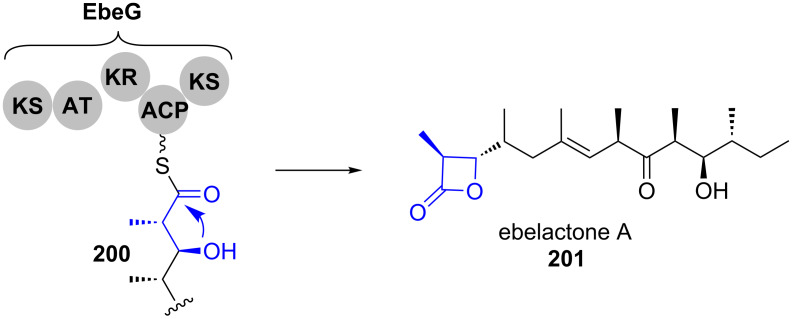
Spontaneous cyclisation leads to off-loading of ebelactone A (**201**) from the PKS machinery [163].

**Ebelactone A.** Ebelactone A (**201**) is an esterase inhibitor of PKS type I origin that is produced by *Streptomyces aburaviensis* ATCC 31 860 [162]. Similar to salinosporamide A (**199**), the respective biosynthetic gene cluster neither encodes a modular nor a lone-standing thioesterase domain. Instead, in vitro studies with the SNAC-thioester bound acyclic intermediate demonstrated the spontaneous heterocyclisation by nucleophilic attack of the β-hydroxy group on the thioester of **200**, resulting in the off-loaded ebelactone A (**201**) (Scheme 28) [163]. The β-lactone moiety of the human pancreatic lipase inhibitor lipstatin is also formed in a similar fashion.

### 2 Nitrogen-containing heterocycles

**Scheme 29:**
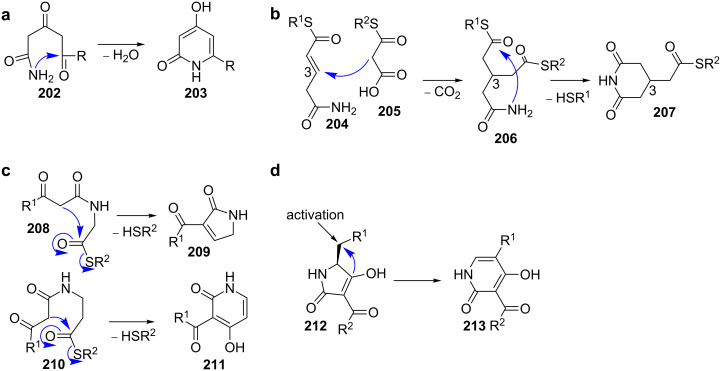
Mechanisms for the formation of nitrogen heterocycles.

Nitrogen-containing heterocycles are established in four principal ways (Scheme 29).

The biosynthesis of pyridinones (**203**, **207**, **211** or **213**) is mechanistically particularly diverse. It occurs via condensation reactions between carbonyl groups and nitrogen-containing functionalities, Michael addition–lactamisation cascades (similar to the mechanism for 4-substituted pyran-2-ones shown in Scheme 2e), Dieckmann condensations as well as oxidative ring expansion of tetramates (**212**, a–d in Scheme 29). Tetramates **209** are formed by Dieckmann condensation (c in Scheme 29).

#### 2.1 Pyridinones

**Scheme 30:**
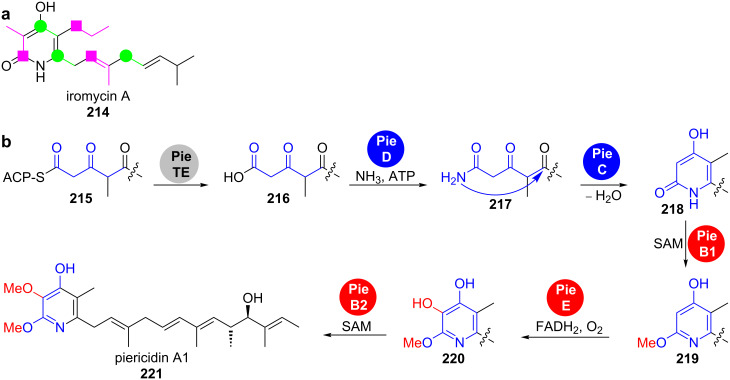
Biosynthesis of highly substituted α-pyridinones. a) Feeding experiments confirmed the polyketide origin of iromycin A (**214**). b) The heterocycle in piericidin A1 (**221**) is formed by condensation between an amide and a ketone [164–166].

**2.1.1 Condensation between carbonyl groups and nitrogen nucleophiles: Piericidin.** Highly substituted α-pyridinones that carry the polyketide chain in the 6-position are assembled by type I PKS. In 2007, Grond et al*.* isolated the iromycins **214** from *Streptomyces bottropensis* sp. Gö Dra 17 and provided initial information on their biosynthesis by feeding studies with istope-labelled precursors (Scheme 30a) [164]. These experiments revealed that all carbon atoms of the heterocycle are derived from acetate or propionate units and that no amino acid is incorporated. The nitrogen thus originates from transamination.

More detailed information about the mechanism became available from the gene cluster analysis of the *O*-methylated, highly substituted α-pyridinone piericidin A1 (**221**) from *Streptomyces piomogeues* var. Hangzhouwanensis (Scheme 30b) [165–166]. Apart from the genes that code for a modular type I PKS as well as *O*-methyltransferases and an oxygenase, the cluster contains *pieC* and *pieD* whose gene products were annotated as a hypothetical protein and an asparagine synthase, respectively.

While inactivation of *pieD* led to complete abolishment of piericidin A1 (**221**) production, inactivation of *pieC* only led to a decrease in titre to about 35–50% of the wild-type levels, suggesting that *pieC* is not essential for biosynthesis [164]. PieC belongs to the SRPBCC superfamily, which has previously been shown to be involved in the controlled cyclisation events catalysed by type II PKS [167–168]. These enzymes have a deep hydrophobic ligand-binding pocket, which templates particular cyclisation patterns.

The fact that PieC was dispensable for piericidin A1 (**221**) biosynthesis was explained by that it could either be complemented by other endogenous cyclases in *S. piomogeues* or that the thermodynamically favoured formation of the six-membered heterocycle occurs spontaneously in the absence of the enzyme.

**Scheme 31:**
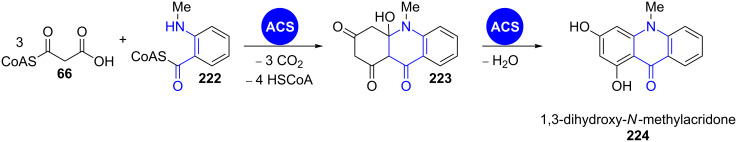
Acridone synthase (ACS) catalyses the formation of 1,3-dihydroxy-*N*-methylacridone (**224**) by condensation of *N*-methylanthraniloyl-CoA (**222**) and three units of malonyl-CoA (**66**) [171].

**Acridones.** The acridones are pyridin-4(1*H*)-one-containing metabolites of *Rutaceae*, which serve for UV protection and antimicrobial defense [169–170]. They are produced by various acridone synthases (ACSs), which are expressed depending on external triggers like irradiation or fungal elicitation (Scheme 31).

ACSs are plant type III PKSs that catalyse condensation between *N*-methylanthraniloyl-CoA (**222**) and three units of malonyl-CoA (**66**) to yield 1,3-dihydroxy-*N*-methylacridone (**224**, Scheme 31). The cyclisation mechanism passes hemiaminal **223** that then undergoes dehydrative aromatisation. ACSs show high similarity on the amino acid level to other type III PKS systems like chalcone synthases and benzalacetone synthases, but their strict substrate specificity for nitrogen-containing starter units avoids mispriming with precursors of the latter group of enzymes [172]. Altering of synthase specificity and thus interconversion into each other has been demonstrated [173].

**2.1.2 Michael addition–lactamisation: Glutarimides.** The biosynthesis of the glutarimides proceeds similar to δ-lactone biosynthesis and has been described in chapter 1.6.

**Scheme 32:**
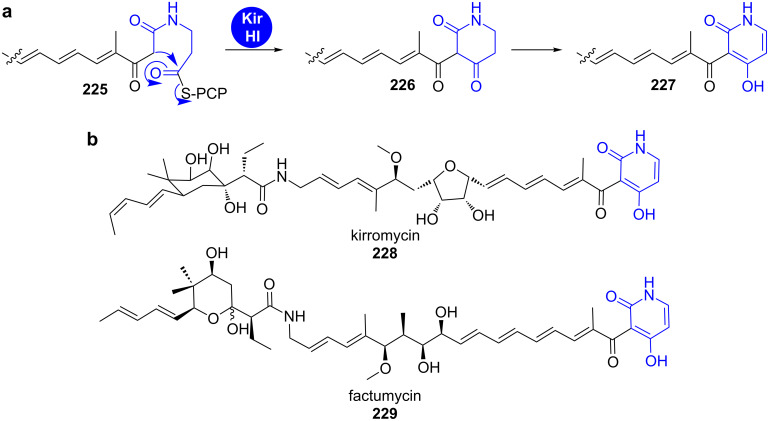
A Dieckmann condensation leads to the formation of a 3-acyl-4-hydroxypyridin-2-one **227** and removes the biosynthetic precursor from the PKS–NRPS hybrid assembly line during kirromycin (**228**) biosynthesis [174].

**2.1.3 Dieckmann condensation:** Actinomycete-derived pyridinone natural products are formed in a similar fashion as tetramates (see chapter 2.2.1) [174]. Elaborate polyketide intermediates are condensed to the amine functionality of a PCP-bound β-alanine on the terminal module of a PKS–NRPS assembly line (Scheme 32). The resulting *N*-β-ketoacyl-β-alanyl-*S*-PCP (3-(3-oxoalkylamido)propanoyl-*S*-PCP, **225**) is then processed by a Dieckmann cyclase to give the heterocycle **226** that tautomerises to the 4-hydroxy-3-acylpyridin-2-one (**227**) [174].

The elfamycin antibiotics kirromycin (**228**) and factumycin (**229**) from *Streptomyces collinus* Tü 365 and WAC5292, respectively, are formed via this mechanism. For kirromycin (**228**) biosynthesis, it has been shown by in vitro activity testing that the Dieckmann cyclase KirHI condenses *N*-acetoacetyl β-alanyl-SNAC as well as *N*-acetoacetyl D-alanyl-SNAC and *N*-acetoacetyl glycyl-SNAC to the corresponding pyridinones and tetramates (see also chapter 2.2.1). The factumycin gene cluster was recently sequenced and contains a close homolog of KirHI, FacHI, which is supposed to catalyse the analogous reaction in its biosynthesis.

In accordance with the release from the assembly line by Dieckmann condensation, both clusters do not contain a TE domain [175]. In contrast, the cluster of the pyridinone-less elfamycin antibiotic L-681217 from *Streptomyces cattleya* does not harbour a Dieckmann cyclase homolog, but a conventional TE domain [175–177].

**2.1.4 Oxidative ring expansion:** The oxidative ring expansion is an alternative biosynthetic strategy that leads to pyridinone rings in fungal systems. The precursors of these expansion reactions are tetramic acids, whose biosynthetic characteristics are highlighted in chapter 2.2.1.

**Scheme 33:**
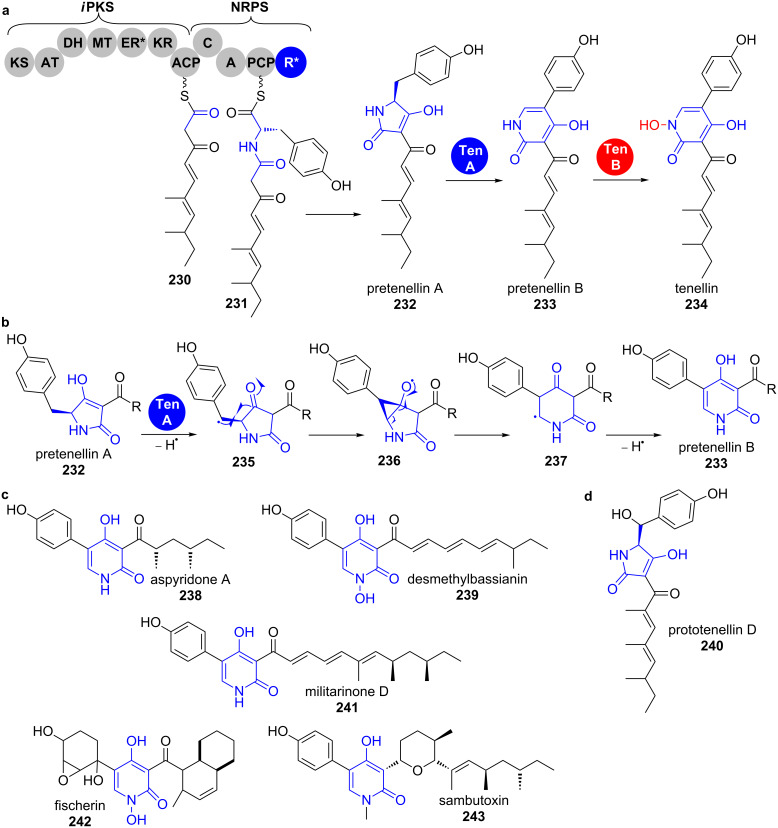
a) Biosynthesis of the pyridinone tenellin (**234**). b) A radical mechanism was proposed for the ring-expansion reaction catalysed by TenA. c) Other fungal pyridinone-containing hybrid *i*PKS–NRPS natural products [178,180].

The backbone of the tenellins from the insect pathogen *Beauveria bassiana* is assembled by an *i*PKS–NRPS hybrid and the resulting *N*-β-ketoacyl-β-tyrosinyl-*S*-PCP intermediate **231** is cyclised by an R* domain to yield the tetramic acid pretenellin A (**232**, Scheme 33a). Two cytochrome P450 monooxygenases then catalyse the consecutive ring expansion to the pyridinone and *N*-hydroxylation. TenA was annotated as the ring expandase responsible for pyridinone formation.

The mechanism of this unusual ring-expansion reaction remains unclear in detail. The authors however presented preliminary indications that point towards a radical mechanism without isolable intermediates (Scheme 33b) [178]. This was supported by the presence of the shunt product prototenellin D (**240**) in the wild-type strain and in several knockout transformants. Conversion experiments with cell-free extracts showed that **240** is not a competent substrate of the tailoring enzymes in the cluster. It was suggested that other oxidising enzymes with appropriate substrate specificity must be encoded in the *Beauveria bassiana* genome and responsible for prototenellin (**240**) formation. A similar situation must be given for compounds **238**, **239** and **241**–**243**, whose clusters contain ring expandase candidates with high identity to TenA and for which similarly hydroxylated metabolites were isolated (Scheme 33c). Other authors suggested mechanisms that pass a quinonemethide intermediate [179].

The *N*-hydroxylation reaction occurring from pretenellin B (**233**) to tenellin (**234**) is catalysed by the second cytochrome P450 monooxygenase TenB. This type of reaction is usually rather catalysed by FAD-dependent monooxygenases and nonheme iron-containing monooxygenases [181–185].

The cytochrome P450 monooxygenase ApdE (48% amino acid identity to TenA) was shown to catalyse a similar ring-expansion reaction in aspyridone A (**238**) biosynthesis (Scheme 33c). This enzyme, however, shows a more diverse oxidation chemistry leading not only to the pyridinone, but also to a β-hydroxytetramic acid as well as a dephenylated product.

**Oxazoles.** Natural products featuring oxazole moieties are predominantly derived from the nucleophilic attack of a serine side chain hydroxy group on a carbonyl carbon of the peptide backbone. This has been shown for the oxazoles in thiazole/oxazole-modified microcins (TOMMs) which are a group of ribosomally synthesised and posttranslationally modified peptides as well as for NRPS-derived natural products [186]. In the case of NRPS, the assembly is accomplished by a modified condensation domain (designated as heterocyclisation domain) and the resulting oxazoline is often subsequently aromatised to the oxazole by a flavin-dependent oxygenase domain [187]. However, some PKS–NRPS derived oxazoles originate from a different biosynthetic route.

**Scheme 34:**
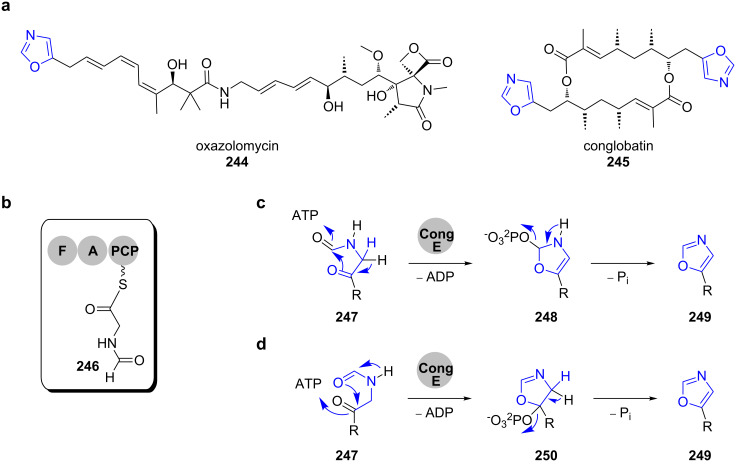
a) Oxazole-containing PKS–NRPS-derived natural products oxazolomycin (**244**) and conglobatin (**245**). b) Formylglycyl-*S*-PCP precursor for oxazole formation. c) and d) Proposed mechanisms for oxazole formation as suggested by Leadlay and co-workers [192].

Oxazolomycin (**244**) is a polyene spiro-linked γ-lactam/β-lacton antibiotic that was originally isolated from *Streptomyces albus* (Scheme 34a) [188–189].

Isotope-labelling studies have shown that instead of serine, three molecules of glycine are incorporated into its carbon backbone. The analysis of the respective biosynthetic gene cluster revealed the absence of canonical heterocyclisation or oxidation domains [190]. Instead, the loading module OzmO contains a formylation domain that transfers the formyl group of formyl-tetrahydrofolate onto glycin-*S*-PCP (Scheme 34b) [191]. The resulting formyl-glycin-*S*-PCP **246** serves as the precursor for cyclisation.

Recently, Leadlay and co-workers proposed a mechanism for oxazole formation in the biosynthesis of the *C*2-symmetrical macrodiolide conglobatin (**245**) that was isolated from *Streptomyces conglobatus* ATCC 31005 [192–193]. In the biosynthetic gene cluster, a putative cyclodehydratase CongE is coded that is homologous to OzmP from the oxazolomycin (**244**) gene cluster. Molecular modelling studies suggested, that CongE belongs to the family of N-type ATP (pyro)phosphohydrolases and contains the conserved ATP-binding motif SGGKDS. In analogy to a mechanism previously reported by Dunbar and co-workers, Leadlay and co-workers hypothesised that CongE promotes oxazole formation by activation of one of the carbonyl amide oxygens by either phosphorylation or adenyl transfer followed by nucleophilic attack and elimination (Scheme 34c and d) [192,194].

#### 2.2 Pyrrolidinones

**Scheme 35:**
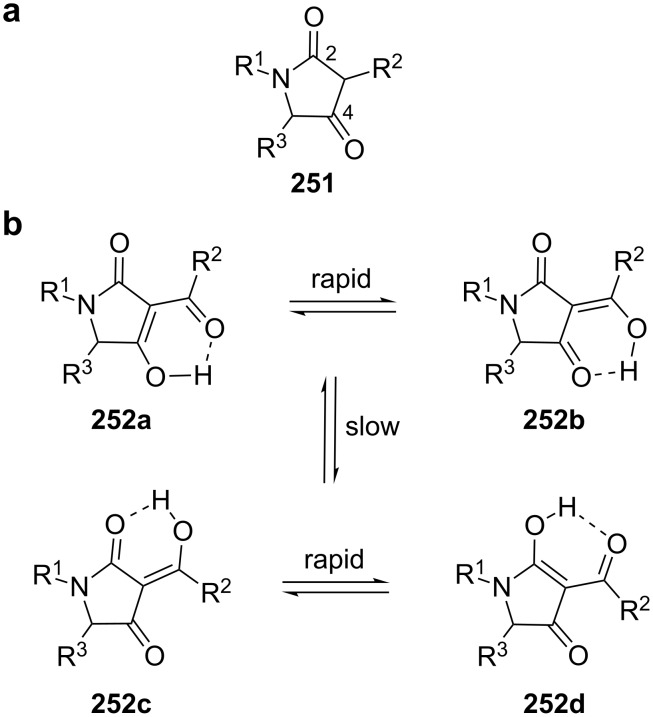
Structure of tetramic acids **251** (a) and major tautomers of 3-acyltetramic acids **252a**–**d** (b). Adapted from [195].

**2.2.1 Dieckmann condensation: Tetramates.** Natural products featuring a tetramic acid moiety (pyrrolidine-2,4-dione **251**, Scheme 35) have been isolated from terrestrial and marine organisms, including fungi, bacteria and sponges. Due to different oxidation states of the five-membered heterocyclic core and diversified downstream processing, tetramates display a high structural complexity.

This chemically rich diversity results in a wide range of biological activities, including antimicrobial, antitumor and antiviral properties [195–199]. The pharmacologically most relevant tetramates are – as in the case of their corresponding oxygen-analogues tetronates (see chapter 1.7) – those featuring 3-acyl residues. Tetramic acids are usually present in their 2,4-diketo form **251** and 3-acyltetramic acids can in principle form nine different tautomers, of which typically four are detectable in solution (**252a**–**d**, Scheme 35b).

Tetramate cores are typically derived from PKS–NRPS hybrid assembly lines, yielding linear 3-(β-ketoamide)propanoyl-thioester intermediates. The subsequent Dieckmann cyclisation releases the tetramate from the megasynthetase. There are basically four different types of enzymatic units responsible for this process, which are described to date: module-embedded R*- and TE-domains, as well as lone-standing PyrD3/PyrD4-homologs and Dieckmann cyclases.

**Scheme 36:**
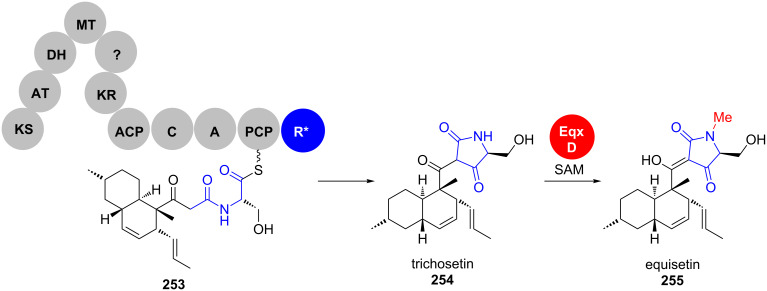
Equisetin biosynthesis. R*: terminal reductive domain. Adapted from [202].

In fungal *i*PKS–NRPS systems, a terminal reductive domain (R*) directly catalyses the tetramate cyclisation without intermediacy of a free aldehyde intermediate [179]. Studies of Schmidt et al. provided the first evidence for this biosynthetic route, utilising the R* domain of the equisetin (**255**) pathway from *Fusarium* strains (Scheme 36) [200–201].

The reaction required no cofactor, despite a conserved N-terminal NAD(P)H binding motif that is characteristic for the SDR superfamily. In addition, phylogenetic analyses revealed that R*-domains represent a distinct branch in the SDR superfamily tree. Subsequent studies showed that the equisetin synthetase genes had been misidentified as the fusaridione A synthetase genes and the cluster was reassigned correctly [202]. Sequence alignments in the same study also identified corresponding R*-domains in the biosynthetic pathways of the spiro-tetramates.

**Scheme 37:**
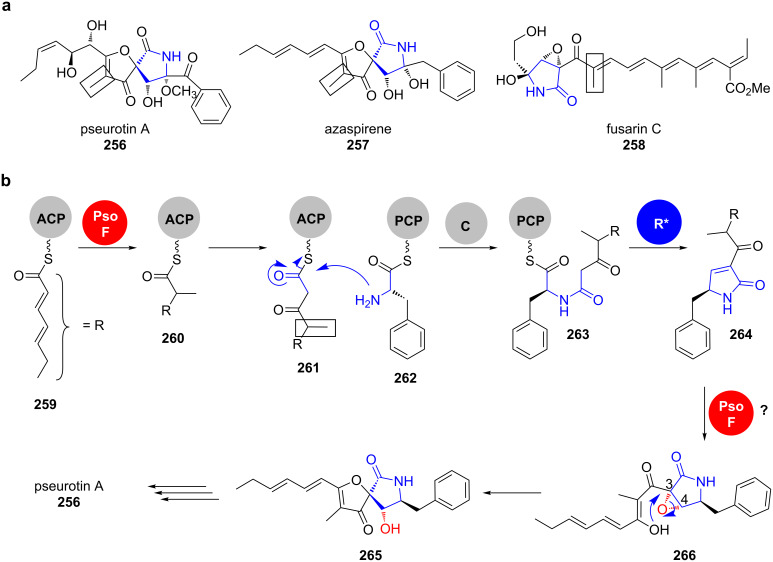
a) Polyketides for which a similar biosynthetic logic was suggested. b) Pseurotin A (**256**) biosynthesis. Modified from [203].

Pseurotins are *Aspergillal* natural products from the group of the 3-spirotetramates, which display a wide array of biological activities (Scheme 37a).

Their spiro centre is installed by epoxidation of the C3–C4 double bond of the tetramate ring in **264** and subsequent epoxide opening by the 3’-enol oxygen of the side chain. Interestingly, it was shown that the bifunctional epoxidase/C-MT PsoF also catalyses a gate-keeping methylation in *trans* on the stage of the nascent tetraketide (Scheme 37, highlighted in boxes). This modification is crucial for the acyl-chain transfer from PKS (**261**) to NRPS (**263**) as well as the epoxidation reaction that yields the final spiro structure in **265** and pseurotin A (**256**). Multiple methylation and oxidation steps give rise to a high chemical diversity in the pseurotin compound family [203–204]. This gate-keeping methylation was also proposed for other fungal tetramates (Scheme 37b).

**Figure 7:**
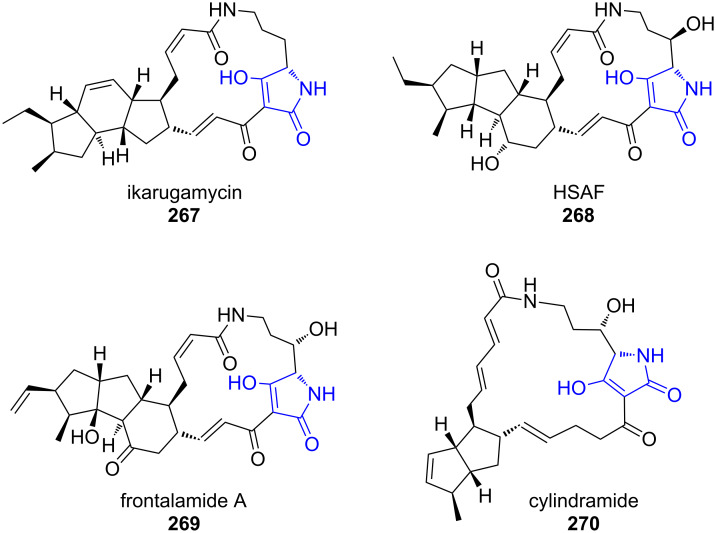
Representative examples of PTMs with varying ring sizes and oxidation patterns [205–206].

For the polycyclic tetramate macrolactams (PTMs), a module-embedded TE domain that belongs to the α/β-hydrolase family adopts the function of the fungal R*-domain [205–206]. The tetramic acid is incorporated into a macrolactam ring, which is fused to a set of two or three carbacycles of varying size, cyclization pattern and oxidation level (Figure 7). This rich structural diversity of PTMs, which are produced in phylogenetically diverse bacteria results in a broad spectrum of biological activities, including compounds with antifungal, antibiotic, and antitumoural properties [207].

**Scheme 38:**
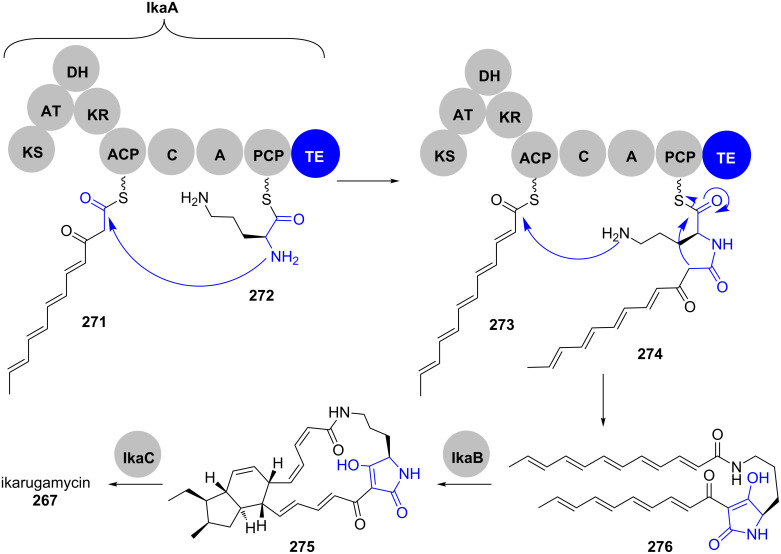
Ikarugamycin biosynthesis. Adapted from [209–211].

Ikarugamycin (**267**) is a PTM produced by various *Streptomyces* species that shows a broad spectrum of biological activity including antimicrobial and cytotoxic properties [208]. Its biosynthesis has been reconstituted in *E. coli* and has shown to be remarkably streamlined, utilising only the three enzymes IkaABC to build up its highly complex structure (Scheme 38) [209–210].

IkaA is a mixed *i*PKS–NRPS, in which the *i*PKS provides two ACP-bound hexaketides **271** and **273**. The condensation domain of the NRPS attaches these two polyketide chains to the amine functionalities of PCP-bound ornithine **272**. The tetramate **276** is released by the TE domain and further processed towards ikarugamycin (**267**) [212].

**Scheme 39:**
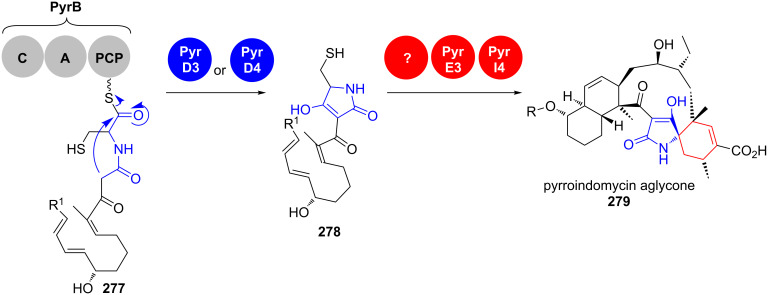
Tetramate formation in pyrroindomycin aglycone (**279**) biosynthesis [213–215].

Isolated in a screening against various drug-resistant pathogens, the pyrroindomycins A and B from *Streptomyces rugosporus* LL-42D005 (NRRL 21084) were the first discovered natural products containing a cyclohexene spiro-linked tetramate moiety combined with a *trans*-dialkyldecalin system in their aglycone (**279**) (Scheme 39) [213–214].

The linear carbon backbone is assembled by the modular PKS type I system PyrA1-A8 in a colinear manner and passed to the NRPS PyrB, which catalyses an aminoacyl extension with L-cysteine [215]. This linear precursor **277** is then cleaved off the megaenzyme by a Dieckmann condensation yielding the tetramate moiety in **278**. Gene deletion experiments and in vitro assays revealed that this reaction is catalysed individually by the two phylogenetically distinct enzymes PyrD3 and PyrD4. Their respective biosynthetic genes are closely clustered and located centrally in the PKS gene cluster. Homologs of PyrD3 and PyrD4 were found in biosynthetic gene clusters of the structurally related spirotetronates. The introduction of *chlD4* from the spirotetronate chlorothricin (**152**) biosynthesis even partially restored pyrroindomycin production in a Δ*pyrD3-D4* deletion mutant, highlighting the similarities in the tetronate/tetramate formation chemistry in these natural products.

**Scheme 40:**
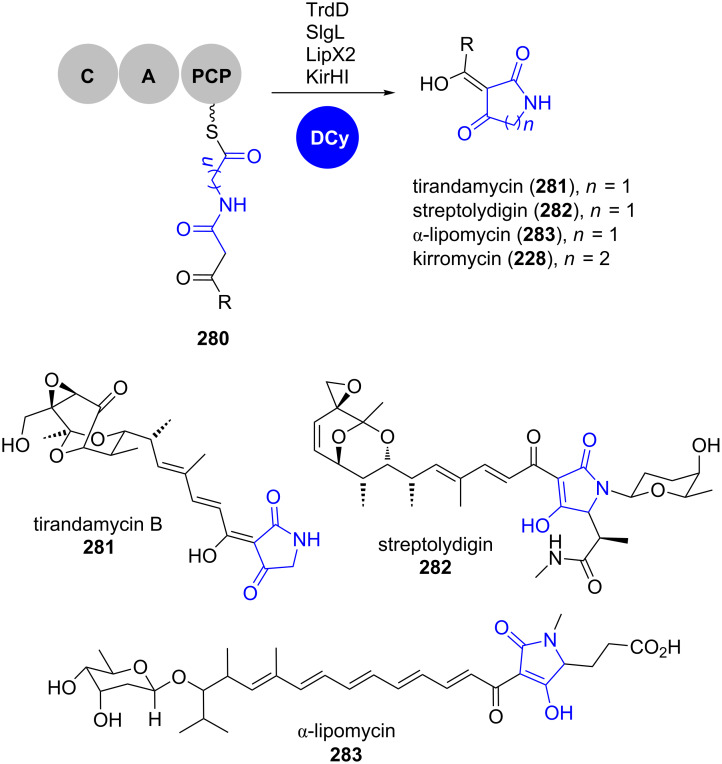
Dieckmann cyclases catalyse tetramate or 2-pyridone formation in the biosynthesis of, for example, tirandamycin B (**281**), streptolyldigin (**282**), α-lipomycin (**283**) and kirromycin (**228**), respectively. DCy: Dieckmann cyclase. Adapted from [174].

Surprisingly, in some actinomycete-derived tetramic acid-containing natural products, R* or TE domains as well as PyrD3/PyrD4-homologs are absent in the respective gene clusters. On the contrary, recent in vitro studies revealed conserved dedicated Dieckmann cyclases as catalysts in the biosynthetic pathways towards tirandamycin B (**281**), streptoylydigin (**282**) and α-lipomycin (**283**), respectively (Scheme 40) [174].

In all these pathways, homologous genes are found directly upstream of the NRPS genes and in their deletion mutants, tetramate formation was abolished. In in vitro reactions, simplified SNAC thioester substrate surrogates were converted into tetramates and mutational studies revealed a conserved catalytic triad consisting of Cys_88_-Asp_115_-His_253_. The respective Dieckmann cyclases are phylogenetically distinct from fungal R* domains, bacterial TE domains and PyrD3/PyrD4. Interestingly, this paradigm also applies for bacterially derived 2-pyridone scaffolds such as kirromycin (**228**) (see chapter 2.1.3).

## Conclusion

Due to their attractive biological activity and abundance, oxygen and nitrogen heterocycles-containing polyketides are highly relevant. Recent years have seen a steady progress in the understanding of their biosynthesis and plenty of novel enzymology has been uncovered in this context. It is now clear that heterocycle formation occurs by an exceptionally broad range of mechanisms. Nevertheless, there is still plenty of room for future studies on the biosynthesis of other types of heterocycles as well as on the catalytic mechanisms and the structures of cyclising enzymes. In principle, all these enzymes also represent future candidates for the development of novel types of biocatalysts for chemoenzymatic synthesis.

## References

[1] Taylor R D, MacCoss M, Lawson A D G (2014). J Med Chem.

[2] Pozharskii A F, Soldatenkov A T, Katritzky A R (2011). Heterocycles in Life and Society: An Introduction to Heterocyclic Chemistry, Biochemistry and Applications.

[3] Dua R, Shrivastava S, Sonwane S K, Shrivastava S K (2011). Adv Biol Res.

[4] Liu T, Cane D E, Deng Z, Hopwood D A (2009). The Enzymology of Polyether Biosynthesis. Complex Enzymes in Microbial Natural Product Biosynthesis, Part B: Polyketides, Aminocoumarins and Carbohydrates.

[5] Fischbach M A, Walsh C T (2006). Chem Rev.

[6] Friedrich S, Hahn F (2015). Tetrahedron.

[7] Lechner H, Pressnitz D, Kroutil W (2015). Biotechnol Adv.

[8] Hertweck C (2009). Angew Chem, Int Ed.

[9] Tang Y, Tsai S-C, Khosla C (2003). J Am Chem Soc.

[10] Weissman K J, Leadlay P F (2005). Nat Rev Microbiol.

[11] Staunton J, Weissman K J (2001). Nat Prod Rep.

[12] Parenty A, Moreau X, Campagne J-M (2006). Chem Rev.

[13] Richter M E A, Traitcheva N, Knüpfer U, Hertweck C (2008). Angew Chem, Int Ed.

[14] Pöplau P, Frank S, Morinaka B I, Piel J (2013). Angew Chem, Int Ed.

[15] Berkhan G, Hahn F (2014). Angew Chem, Int Ed.

[16] Sudek S, Lopanik N B, Waggoner L E, Hildebrand M, Anderson C, Liu H, Patel A, Sherman D H, Haygood M G (2007). J Nat Prod.

[17] Piel J (2002). Proc Natl Acad Sci U S A.

[18] Luhavaya H, Dias M V B, Williams S R, Hong H, de Oliveira L G, Leadlay P F (2015). Angew Chem, Int Ed.

[19] Bieber B, Nüske J, Ritzau M, Gräfe U (1998). J Antibiot.

[20] Naruse N, Goto M, Watanabe Y, Terasawa T, Dobashi K (1998). J Antibiot.

[21] Metsä-Ketelä M, Oja T, Taguchi T, Okamoto S, Ichinose K (2013). Curr Opin Chem Biol.

[22] Das A, Khosla C (2009). Acc Chem Res.

[23] Malpartida F, Hopwood D A (1984). Nature.

[24] Fernández-Moreno M A, Martínez E, Boto L, Hopwood D A, Malpartida F (1992). J Biol Chem.

[25] Keatinge-Clay A T, Maltby D A, Medzihradszky K F, Khosla C, Stroud R M (2004). Nat Struct Mol Biol.

[26] McDaniel R, Ebert-Khosla S, Hopwood D A, Khosla C (1993). J Am Chem Soc.

[27] Taguchi T, Kunieda K, Takeda-Shitaka M, Takaya D, Kawano N, Kimberley M R, Booker-Milburn K I, Stephenson G R, Umeyama H, Ebizuka Y (2004). Bioorg Med Chem.

[28] Caballero J L, Martinez E, Malpartida F, Hopwood D A (1991). MGG, Mol Gen Genet.

[29] Cole S P, Rudd B A, Hopwood D A, Chang C-J, Floss H G (1987). J Antibiot.

[30] He Q, Li L, Yang T, Li R, Li A (2015). PLoS One.

[31] Itoh T, Taguchi T, Kimberley M R, Booker-Milburn K I, Stephenson G R, Ebizuka Y, Ichinose K (2007). Biochemistry.

[32] Taguchi T, Ebizuka Y, Hopwood D A, Ichinose K (2001). J Am Chem Soc.

[33] Grocholski T, Oja T, Humphrey L, Mäntsälä P, Niemi J, Metsä-Ketelä M (2012). J Bacteriol.

[34] Oja T, Palmu K, Lehmussola H, Leppäranta O, Hännikäinen K, Niemi J, Mäntsälä P, Metsä-Ketelä M (2008). Chem Biol.

[35] Oja T, Klika K D, Appassamy L, Sinkkonen J, Mäntsälä P, Niemi J, Metsä-Ketelä M (2012). Proc Natl Acad Sci U S A.

[36] Oja T, Niiranen L, Sandalova T, Klika K D, Niemi J, Mäntsälä P, Schneider G, Metsä-Ketelä M (2013). Proc Natl Acad Sci U S A.

[37] Fuller A T, Mellows G, Woolford M, Banks G T, Barrow K D, Chain E B (1971). Nature.

[38] Chain E B, Mellows G (1974). J Chem Soc, Chem Commun.

[39] Chain E B, Mellows G (1977). J Chem Soc, Perkin Trans 1.

[40] Clayton J P, O’Hanlon P J, Rogers N H (1980). Tetrahedron Lett.

[41] Shiozawa H, Shimada A, Takahashi S (1997). J Antibiot.

[42] Hughes J, Mellows G (1978). Biochem J.

[43] Hughes J, Mellows G (1978). J Antibiot.

[44] Hughes J, Mellows G (1980). Biochem J.

[45] El-Sayed A K, Hothersall J, Cooper S M, Stephens E, Simpson T J, Thomas C M (2003). Chem Biol.

[46] Gao S-S, Hothersall J, Wu J, Murphy A C, Song Z, Stephens E R, Thomas C M, Crump M P, Cox R J, Simpson T J (2014). J Am Chem Soc.

[47] Cooper S M, Laosripaiboon W, Rahman A S, Hothersall J, El-Sayed A K, Winfield C, Crosby J, Cox R J, Simpson T J, Thomas C M (2005). Chem Biol.

[48] Hothersall J, Wu J, Rahman A S, Shields J A, Haddock J, Johnson N, Cooper S M, Stephens E R, Cox R J, Crosby J (2007). J Biol Chem.

[49] Cooper S M, Cox R J, Crosby J, Crump M P, Hothersall J, Laosripaiboon W, Simpson T J, Thomas C M (2005). Chem Commun.

[50] Julien B, Tian Z-Q, Reid R, Reeves C D (2006). Chem Biol.

[51] Nicolaou K C, Prasad C V C, Somers P K, Hwang C K (1989). J Am Chem Soc.

[52] Nicolaou K C, Prasad C V C, Somers P K, Hwang C K (1989). J Am Chem Soc.

[53] Shen B, Kwon H-J (2002). Chem Rec.

[54] Nelson M E, Priestley N D (2002). J Am Chem Soc.

[55] Ashworth D M, Robinson J A, Turner D L (1988). J Chem Soc, Perkin Trans 1.

[56] Woo A J, Strohl W R, Priestley N D (1999). Antimicrob Agents Chemother.

[57] Earle M J, Priestley N D (1997). Bioorg Med Chem Lett.

[58] Rong J, Nelson M E, Kusche B, Priestley N D (2010). J Nat Prod.

[59] Smith W C, Xiang L, Shen B (2000). Antimicrob Agents Chemother.

[60] Rebets Y, Brötz E, Manderscheid N, Tokovenko B, Myronovskyi M, Metz P, Petzke L, Luzhetskyy A (2015). Angew Chem, Int Ed.

[61] Matilla M A, Stöckmann H, Leeper F J, Salmond G P C (2012). J Biol Chem.

[62] Müller M, Kusebauch B, Liang G, Beaudry C M, Trauner D, Hertweck C (2006). Angew Chem, Int Ed.

[63] He J, Müller M, Hertweck C (2004). J Am Chem Soc.

[64] He J, Hertweck C (2003). Chem Biol.

[65] He J, Hertweck C (2004). J Am Chem Soc.

[66] Werneburg M, Busch B, He J, Richter M E A, Xiang L, Moore B S, Roth M, Dahse H-M, Hertweck C (2010). J Am Chem Soc.

[67] Werneburg M, Hertweck C (2008). ChemBioChem.

[68] Henrot M, Richter M E A, Maddaluno J, Hertweck C, De Paolis M (2012). Angew Chem, Int Ed.

[69] Bode H B, Wenzel S C, Irschik H, Höfle G, Müller R (2004). Angew Chem, Int Ed.

[70] Kopp M, Irschik H, Gemperlein K, Buntin K, Meiser P, Weissman K J, Bode H B, Müller R (2011). Mol BioSyst.

[71] Winkler A, Łyskowski A, Riedl S, Puhl M, Kutchan T M, Macheroux P, Gruber K (2008). Nat Chem Biol.

[72] Winkler A, Motz K, Riedl S, Puhl M, Macheroux P, Gruber K (2009). J Biol Chem.

[73] Winkler A, Hartner F, Kutchan T M, Glieder A, Macheroux P (2006). J Biol Chem.

[74] Gaweska H M, Roberts K M, Fitzpatrick P F (2012). Biochemistry.

[75] Guo C-J, Wang C C C (2014). Front Microbiol.

[76] Chiang Y-M, Szewczyk E, Davidson A D, Keller N, Oakley B R, Wang C C C (2009). J Am Chem Soc.

[77] Chiang Y-M, Oakley C E, Ahuja M, Entwistle R, Schultz A, Chang S-L, Sung C T, Wang C C C, Oakley B R (2013). J Am Chem Soc.

[78] Yu J, Chang P-K, Ehrlich K C, Cary J W, Bhatnagar D, Cleveland T E, Payne G A, Linz J E, Woloshuk C P, Bennett J W (2004). Appl Environ Microbiol.

[79] Ehrlich K C, Yu J, Cotty P J (2005). J Appl Microbiol.

[80] Asao T, Büchi G, Abdel-Kader M M, Chang S B, Wick E L, Wogan G N (1965). J Am Chem Soc.

[81] Holzapfel C W, Steyn P S, Purchase I F H (1966). Tetrahedron Lett.

[82] Cox R (2014). Nat Prod Rep.

[83] Minto R E, Townsend C A (1997). Chem Rev.

[84] Townsend C A (2014). Nat Prod Rep.

[85] Crawford J M, Thomas P M, Scheerer J R, Vagstad A L, Kelleher N L, Townsend C A (2008). Science.

[86] Watanabe C M H, Townsend C A (2002). Chem Biol.

[87] Sakuno E, Yabe K, Nakajima H (2003). Appl Environ Microbiol.

[88] Sakuno E, Wen Y, Hatabayashi H, Arai H, Aoki C, Yabe K, Nakajima H (2005). Appl Environ Microbiol.

[89] Simpson T J, de Jesus A E, Steyn P S, Vleggaar R (1982). J Chem Soc, Chem Commun.

[90] Townsend C A, Christensen S B, Davis S G (1982). J Am Chem Soc.

[91] Townsend C A, Christensen S B (1985). J Am Chem Soc.

[92] Townsend C A, Christensen S B, Davis S G (1988). J Chem Soc, Perkin Trans 1.

[93] Wen Y, Hatabayashi H, Arai H, Kitamoto H K, Yabe K (2005). Appl Environ Microbiol.

[94] McGuire S M, Townsend C A (1993). Bioorg Med Chem Lett.

[95] Chang P-K, Yabe K, Yu J (2004). Appl Environ Microbiol.

[96] Lin B-K, Anderson J A (1992). Arch Biochem Biophys.

[97] Yabe K, Matsuyama Y, Ando Y, Nakajima H, Hamasaki T (1993). Appl Environ Microbiol.

[98] McGuire S M, Silva J C, Casillas E G, Townsend C A (1996). Biochemistry.

[99] Silva J C, Minto R E, Barry C E, Holland K A, Townsend C A (1996). J Biol Chem.

[100] Conradt D, Schätzle M A, Haas J, Townsend C A, Müller M (2015). J Am Chem Soc.

[101] Henry K M, Townsend C A (2005). J Am Chem Soc.

[102] Cary J W, Ehrlich K C, Bland J M, Montalbano B G (2006). Appl Environ Microbiol.

[103] Ehrlich K C, Montalbano B, Boué S M, Bhatnagar D (2005). Appl Environ Microbiol.

[104] Yabe K, Matsushima K, Koyama T, Hamasaki T (1998). Appl Environ Microbiol.

[105] Prieto R, Woloshuk C P (1997). Appl Environ Microbiol.

[106] Udwary D W, Casillas L K, Townsend C A (2002). J Am Chem Soc.

[107] Townsend C A, Christensen S B, Davis S G (1982). J Am Chem Soc.

[108] Yabe K, Hamasaki T (1993). Appl Environ Microbiol.

[109] Liu T, Cane D E, Deng Z (2009). Methods Enzymol.

[110] Bhatt A, Stark C B W, Harvey B M, Gallimore A R, Demydchuk Y A, Spencer J B, Staunton J, Leadlay P F (2005). Angew Chem, Int Ed.

[111] Gallimore A R, Stark C B W, Bhatt A, Harvey B M, Demydchuk Y, Bolanos-Garcia V, Fowler D J, Staunton J, Leadlay P F, Spencer J B (2006). Chem Biol.

[112] Shiraiwa K, Yuan S, Fujiyama A, Matsuo Y, Tanaka T, Jiang Z-H, Kouno I (2012). J Nat Prod.

[113] Smith A B, Dong S, Brenneman J B, Fox R J (2009). J Am Chem Soc.

[114] Moore R E, Scheuer P J (1971). Science.

[115] Steyn P S, Vleggaar R (1985). J Chem Soc, Chem Commun.

[116] Mao X-M, Zhan Z-J, Grayson M N, Tang M-C, Xu W, Li Y-Q, Yin W-B, Lin H-C, Chooi Y-H, Houk K N (2015). J Am Chem Soc.

[117] Huang J-m, Yokoyama R, Yang C-s, Fukuyama Y (2000). Tetrahedron Lett.

[118] Inoue M, Lee N, Kasuya S, Sato T, Hirama M, Moriyama M, Fukuyama Y (2007). J Org Chem.

[119] Holton R A, Somoza C, Kim H B, Liang F, Biediger R J, Boatman P D, Shindo M, Smith C C, Kim S (1994). J Am Chem Soc.

[120] Holton R A, Kim H B, Somoza C, Liang F, Biediger R J, Boatman P D, Shindo M, Smith C C, Kim S (1994). J Am Chem Soc.

[121] Wender P A, Badham N F, Conway S P, Floreancig P E, Glass T E, Gränicher C, Houze J B, Jänichen J, Lee D, Marquess D G (1997). J Am Chem Soc.

[122] Wender P A, Badham N F, Conway S P, Floreancig P E, Glass T E, Houze J B, Krauss N E, Lee D, Marquess D G, McGrane P L (1997). J Am Chem Soc.

[123] Montemiglio L C, Parisi G, Scaglione A, Sciara G, Savino C, Vallone B (2016). Biochim Biophys Acta, Gen Subj.

[124] Nagano S, Li H, Shimizu H, Nishida C, Ogura H, Ortiz de Montellano P R, Poulos T L (2003). J Biol Chem.

[125] Ogura H, Nishida C R, Hoch U R, Perera R, Dawson J H, Ortiz de Montellano P R (2004). Biochemistry.

[126] Schäberle T F (2016). Beilstein J Org Chem.

[127] Palaniappan N, Alhamadsheh M M, Reynolds K A (2008). J Am Chem Soc.

[128] Hu Z, Reid R, Gramajo H (2005). J Antibiot.

[129] Liu X-j, Kong R-x, Niu M-s, Qiu R, Tang L (2013). J Nat Prod.

[130] Bretschneider T, Heim J B, Heine D, Winkler R, Busch B, Kusebauch B, Stehle T, Zocher G, Hertweck C (2013). Nature.

[131] Heine D, Bretschneider T, Sundaram S, Hertweck C (2014). Angew Chem, Int Ed.

[132] Heine D, Sundaram S, Bretschneider T, Hertweck C (2015). Chem Commun.

[133] Sundaram S, Heine D, Hertweck C (2015). Nat Chem Biol.

[134] Piel J, Hertweck C, Shipley P R, Hunt D M, Newman M S, Moore B S (2000). Chem Biol.

[135] Teufel R, Miyanaga A, Michaudel Q, Stull F, Louie G, Noel J P, Baran P S, Palfey B, Moore B S (2013). Nature.

[136] Cheng Q, Xiang L, Izumikawa M, Meluzzi D, Moore B S (2007). Nat Chem Biol.

[137] Teufel R, Stull F, Meehan M J, Michaudel Q, Dorrestein P C, Palfey B, Moore B S (2015). J Am Chem Soc.

[138] Tao W, Zhu M, Deng Z, Sun Y (2013). Sci China: Chem.

[139] Vieweg L, Reichau S, Schobert R, Leadlay P F, Süssmuth R D (2014). Nat Prod Rep.

[140] Lacoske M H, Theodorakis E A (2015). J Nat Prod.

[141] Demydchuk Y, Sun Y, Hong H, Staunton J, Spencer J B, Leadlay P F (2008). ChemBioChem.

[142] Sun Y, Hahn F, Demydchuk Y, Chettle J, Tosin M, Osada H, Leadlay P F (2010). Nat Chem Biol.

[143] Kanchanabanca C, Tao W, Hong H, Liu Y, Hahn F, Samborskyy M, Deng Z, Sun Y, Leadlay P F (2013). Angew Chem, Int Ed.

[144] Sun Y, Hong H, Gillies F, Spencer J B, Leadlay P F (2008). ChemBioChem.

[145] Wu L-F, He H-Y, Pan H-X, Han L, Wang R, Tang G-L (2014). Org Lett.

[146] Hashimoto T, Hashimoto J, Teruya K, Hirano T, Shin-ya K, Ikeda H, Liu H-w, Nishiyama M, Kuzuyama T (2015). J Am Chem Soc.

[147] Tian Z, Sun P, Yan Y, Wu Z, Zheng Q, Zhou S, Zhang H, Yu F, Jia X, Chen D (2015). Nat Chem Biol.

[148] Tao W, Yurkovich M E, Wen S, Lebe K E, Samborskyy M, Liu Y, Yang A, Liu Y, Ju Y, Deng Z (2016). Chem Sci.

[149] Harborne J B (1993). The Flavonoids: Advances in Research Since 1986.

[150] Vaughn K C, Lax A R, Duke S O (1988). Physiol Plant.

[151] Mayer A M (1986). Phytochemistry.

[152] Sato T, Nakayama T, Kikuchi S, Fukui Y, Yonekura-Sakakibara K, Ueda T, Nishino T, Tanaka Y, Kusumi T (2001). Plant Sci.

[153] Nakayama T, Yonekura-Sakakibara K, Sato T, Kikuchi S, Fukui Y, Fukuchi-Mizutani M, Ueda T, Nakao M, Tanaka Y, Kusumi T (2000). Science.

[154] Nakayama T, Sato T, Fukui Y, Yonekura-Sakakibara K, Hayashi H, Tanaka Y, Kusumi T, Nishino T (2001). FEBS Lett.

[155] Lin Z, Zachariah M M, Marett L, Hughen R W, Teichert R W, Concepcion G P, Haygood M G, Olivera B M, Light A R, Schmidt E W (2014). J Nat Prod.

[156] Raistrick H, Smith G (1936). Biochem J.

[157] Fujii I, Iijima H, Tsukita S, Ebizuka Y, Sankawa U (1987). J Biochem.

[158] Huang K, Yoshida Y, Mikawa K, Fujii I, Ebizuka Y, Sankawa U (1996). Biol Pharm Bull.

[159] Chooi Y-H, Cacho R, Tang Y (2010). Chem Biol.

[160] Udwary D W, Zeigler L, Asolkar R N, Singan V, Lapidus A, Fenical W, Jensen P R, Moore B S (2007). Proc Natl Acad Sci U S A.

[161] Gulder T A M, Moore B S (2010). Angew Chem, Int Ed.

[162] Umezawa H, Aoyagi T, Uotani K, Hamada M, Takeuchi T, Takahashi S (1980). J Antibiot.

[163] Wyatt M A, Ahilan Y, Argyropoulos P, Boddy C N, Magarvey N A, Harrison P H M (2013). J Antibiot.

[164] Surup F, Wagner O, von Frieling J, Schleicher M, Oess S, Müller P, Grond S (2007). J Org Chem.

[165] Liu Q, Yao F, Chooi Y H, Kang Q, Xu W, Li Y, Shao Y, Shi Y, Deng Z, Tang Y (2012). Chem Biol.

[166] Chen Y, Zhang W, Zhu Y, Zhang Q, Tian X, Zhang S, Zhang C (2014). Org Lett.

[167] Zhang W, Tang Y, Hopwood D A (2009). In Vitro Analysis of Type II Polyketide Synthase. Complex Enzymes in Microbial Natural Product Biosynthesis, Part B: Polyketides, Aminocoumarins and Carbohydrates.

[168] Kendrew S G, Katayama K, Deutsch E, Madduri K, Hutchinson C R (1999). Biochemistry.

[169] Maier W, Baumert A, Schumann B, Furukawa H, Gröger D (1993). Phytochemistry.

[170] Junghanns K T, Kneusel R E, Baumert A, Maier W, Gröger D, Matern U (1995). Plant Mol Biol.

[171] Lukačin R, Springob K, Urbanke C, Ernwein C, Schröder G, Schröder J, Matern U (1999). FEBS Lett.

[172] Lukačin R, Schreiner S, Silber K, Matern U (2005). Phytochemistry.

[173] Lukačin R, Schreiner S, Matern U (2001). FEBS Lett.

[174] Gui C, Li Q, Mo X, Qin X, Ma J, Ju J (2015). Org Lett.

[175] Thaker M N, García M, Koteva K, Waglechner N, Sorensen D, Medina R, Wright G D (2012). MedChemComm.

[176] Brötz E, Kulik A, Vikineswary S, Lim C-T, Tan G Y A, Zinecker H, Imhoff J F, Paululat T, Fiedler H-P (2011). J Antibiot.

[177] Kempf A J, Wilson K E, Hensens O D, Monaghan R L, Zimmerman S B, Dulaney E L (1986). J Antibiot.

[178] Halo L M, Heneghan M N, Yakasai A A, Song Z, Williams K, Bailey A M, Cox R J, Lazarus C M, Simpson T J (2008). J Am Chem Soc.

[179] Boettger D, Hertweck C (2013). ChemBioChem.

[180] Schmidt K, Riese U, Li Z, Hamburger M (2003). J Nat Prod.

[181] Rydberg P, Ryde U, Olsen L (2008). J Chem Theory Comput.

[182] Choi Y S, Zhang H, Brunzelle J S, Nair S K, Zhao H (2008). Proc Natl Acad Sci U S A.

[183] Krithika R, Marathe U, Saxena P, Ansari M Z, Mohanty D, Gokhale R S (2006). Proc Natl Acad Sci U S A.

[184] Yamada O, Nan S N, Akao T, Tominaga M, Watanabe H, Satoh T, Enei H, Akita O (2003). J Biosci Bioeng.

[185] Lee J, Simurdiak M, Zhao H (2005). J Biol Chem.

[186] Metelev M V, Ghilarov D A (2014). Mol Biol.

[187] Roy R S, Gehring A M, Milne J C, Belshaw P J, Walsh C T (1999). Nat Prod Rep.

[188] Mori T, Takahashi K, Kashiwabara M, Uemura D, Katayama C, Iwadare S, Shizuri Y, Mitomo R, Nakano F, Matsuzaki A (1985). Tetrahedron Lett.

[189] Gräfe U, Kluge H, Thiericke R (1992). Liebigs Ann Chem.

[190] Zhao C, Ju J, Christenson S D, Smith W C, Song D, Zhou X, Shen B, Deng Z (2006). J Bacteriol.

[191] Zhao C, Coughlin J M, Ju J, Zhu D, Wendt-Pienkowski E, Zhou X, Wang Z, Shen B, Deng Z (2010). J Biol Chem.

[192] Zhou Y, Murphy A C, Samborskyy M, Prediger P, Dias L C, Leadlay P F (2015). Chem Biol.

[193] Westley J W, Liu C-M, Evans R H, Blount J F (1979). J Antibiot.

[194] Dunbar K L, Chekan J R, Cox C L, Burkhart B J, Nair S K, Mitchell D A (2014). Nat Chem Biol.

[195] Schobert R, Schlenk A (2008). Bioorg Med Chem.

[196] Mo X, Li Q, Ju J (2014). RSC Adv.

[197] Ghisalberti E L, Atta-ur-Rahman (2003). Bioactive tetramic acid metabolites. Biactive Natural Products (Part I).

[198] Gossauer A, Herz W, Falk H, Kirby G W (2003). Monopyrrolic Natural Compounds Including Tetramic Acid Derivatives.

[199] Royles B J L (1995). Chem Rev.

[200] Sims J W, Schmidt E W (2008). J Am Chem Soc.

[201] Singh S B, Zink D L, Goetz M A, Dombrowski A W, Polishook J D, Hazuda D J (1998). Tetrahedron Lett.

[202] Kakule T B, Sardar D, Lin Z, Schmidt E W (2013). ACS Chem Biol.

[203] Zou Y, Xu W, Tsunematsu Y, Tang M, Watanabe K, Tang Y (2014). Org Lett.

[204] Tsunematsu Y, Fukutomi M, Saruwatari T, Noguchi H, Hotta K, Tang Y, Watanabe K (2014). Angew Chem, Int Ed.

[205] Du L, Lou L (2010). Nat Prod Rep.

[206] Lou L, Qian G, Xie Y, Hang J, Chen H, Zaleta-Rivera K, Li Y, Shen Y, Dussault P H, Liu F (2011). J Am Chem Soc.

[207] Blodgett J A V, Oh D-C, Cao S, Currie C R, Kolter R, Clardy J (2010). Proc Natl Acad Sci U S A.

[208] Jomon K, Kuroda Y, Ajisaka M, Sakai H (1972). J Antibiot.

[209] Zhang G, Zhang W, Zhang Q, Shi T, Ma L, Zhu Y, Li S, Zhang H, Zhao Y-L, Shi R (2014). Angew Chem, Int Ed.

[210] Antosch J, Schaefers F, Gulder T A M (2014). Angew Chem, Int Ed.

[211] Greunke C, Antosch J, Gulder T A M (2015). Chem Commun.

[212] Li Y, Chen H, Ding Y, Xie Y, Wang H, Cerny R L, Shen Y, Du L (2014). Angew Chem, Int Ed.

[213] Ding W, Williams D R, Northcote P, Siegel M M, Tsao R, Ashcroft J, Morton G O, Alluri M, Abbanat D, Maiese W M (1994). J Antibiot.

[214] Singh M P, Petersen P J, Jacobus N V, Mroczenski-Wildey M J, Maiese W M, Greenstein M, Steinberg D A (1994). J Antibiot.

[215] Wu Q, Wu Z, Qu X, Liu W (2012). J Am Chem Soc.

